# Unified and vector theory of Raman scattering in gas-filled hollow-core fiber across temporal regimes

**DOI:** 10.1063/5.0189749

**Published:** 2024-03-14

**Authors:** Yi-Hao Chen, Frank Wise

**Affiliations:** School of Applied and Engineering Physics, Cornell University, Ithaca, New York 14853, USA

## Abstract

Raman scattering has found renewed interest owing to the development of gas-filled hollow-core fibers, which constitute a unique platform for exploration of novel ultrafast nonlinear phenomena beyond conventional solid-core-fiber and free-space systems. Much progress has been made through models for particular interaction regimes, which are delineated by the relation of the excitation pulse duration to the time scales of the Raman response. However, current experimental settings are not limited to one regime, prompting the need for tools spanning multiple regimes. Here, we present a theoretical framework that accomplishes this goal. The theory allows us to review recent progress with a fresh perspective, makes new connections between distinct temporal regimes of Raman scattering, and reveals new degrees of freedom for controlling Raman physics. Specific topics that are addressed include transient Raman gain, the interplay of electronic and Raman nonlinearities in short-pulse propagation, and interactions of short pulses mediated by phonon waves. The theoretical model also accommodates vector effects, which have been largely neglected in prior works on Raman scattering in gases. The polarization dependence of transient Raman gain and vector effects on pulse interactions via phonon waves is investigated with the model. Throughout this Perspective, theoretical results are compared to the results of realistic numerical simulations. The numerical code that implements the new theory is freely available. We hope that the unified theoretical framework and numerical tool described here will accelerate the exploration of new Raman-scattering phenomena and enable new applications.

## I. INTRODUCTION

Raman scattering is a type of inelastic scattering in which photons undergo frequency up- or down-conversion through interactions with material vibrational or rotational excitations (phonons). It was predicted by Smekal in 1923^1^ and observed experimentally in liquids by Raman and Krishnan in 1928^2–4^ and independently in quartz crystals by Mandelstam and Landsherg during the same period.^5–7^ Raman was awarded the 1930 Nobel prize, although the full and undivided recognition of his contribution has been contentious.^8–11^ There was even a suggested designation of the phenomenon as “the effect of Raman, Mandelstam, and Landsherg.”^12^ Within two years, its discovery had led to numerous subsequent observations in 60 different liquids and gases, which were recognized as providing support for the correctness of the then-new quantum theory.^13^ Moreover, it has given rise to a diverse range of applications. For example, Raman spectroscopy enables identification and characterization of various materials,^14–17^ and Raman amplifiers and Raman lasers extend wavelengths beyond inherent limitations of natural lasing media.^18–25^

With the advent of hollow-core fibers (HCFs), studies of optical interactions with atomic or molecular gases have attracted much attention.^26–30^ In particular, Raman scattering in molecular gases^31–35^ has experienced a resurgence in popularity since the pioneering work by Benabid *et al.*^36^ HCFs offer a substantial reduction of the Raman threshold through the combination of long interaction length and high intensity. Raman frequency down-conversion in H_2_,^37–45^ D_2_,^46–52^ CH_4_,^53–59^ CO_2_,^60,61^ and N_2_^62^ has been demonstrated to generate a wide range of colors. Similarly, frequency up-conversion has been achieved with control of wave-vector matching^63–65^ and successfully applied in the generation of quantum-state-preserving photons.^66^ Further tunability of the converted wavelengths has been realized through the Raman-induced soliton self-frequency shift (SSFS)^67–69^ or its combination with photoionization-induced blue-shifting of the spectrum.^70,71^ Raman-enhanced supercontinuum generation has been demonstrated to cover from vacuum ultraviolet to near-infrared wavelengths,^72–76^ which makes possible pulse compression down to few-cycle durations.^77–82^

**FIG. 1.**
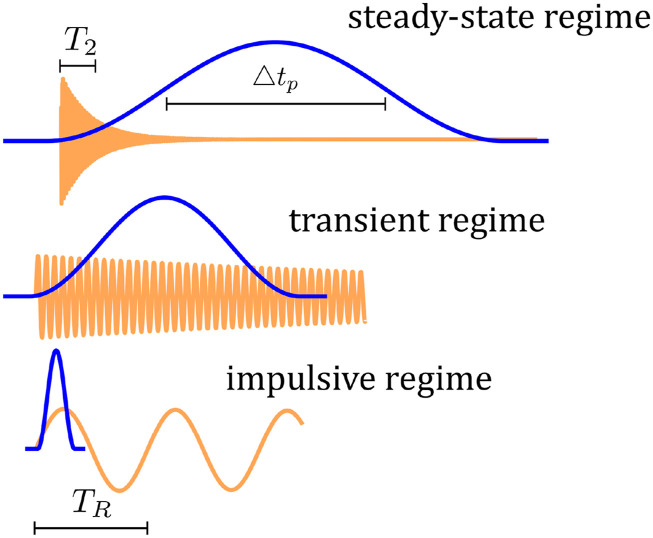
Various Raman regimes depending on the temporal relation between the pulse (blue lines) and the Raman response (orange lines).

The analysis of stimulated Raman scattering (SRS) is commonly classified into multiple regimes based on the relationship of the excitation pulse duration to the material response time (Fig. 1). The most extensively examined regime is the “steady-state” regime, in which the pulse duration (△*t*_*p*_) is significantly longer than the phonon dephasing time *T*_2_.^25,83–85^ In this regime, the Raman response of a medium depends on each temporal segment of a pulse, so the Raman interaction is determined by the pulse’s instantaneous intensity. Moreover, the independence of the temporal segments leads to an incoherent Raman pulse that originates from noise. If the pulse duration becomes shorter than the dephasing time, the interaction enters the “transient” regime. This regime allows long-lasting phonon interactions to drive the SRS process within a pulse. When the Raman gain is high and saturated, “Raman memory” enables coherent generation of Raman pulses that tend to exhibit the same temporal phase profile as the pump pulse.^53^ Ultrashort femtosecond Raman pulses can also be produced without Raman spectral narrowing by scattering the pulse with existing phonons.^86–90^ If the pulse is even shorter than the phonon oscillation period *T*_*R*_ {equivalently, the inverse of the Raman transition frequency $\left[\omega_{R}/\left(2\pi \right)\right]$}, phonons are “impulsively” excited^91^ and persist in the medium after the pulse has left. The excited phonons allow for nonlocal interactions between the first and subsequent excitation pulses, which leads to controllable nonlinear dynamics, such as blue-shifting, red-shifting, and pulse compression, with varying delays between the pulses.^92–95^ By probing the nuclear motions (phonons), the ultrafast dynamics of molecules can be monitored, which underlies time-resolved Raman spectroscopy.^96–100^ In addition, so-called time crystals formed by periodic phonon waves establish an analogy with condense-matter physics, where Bloch oscillations and Zener tunneling are observed.^101^ Generalization of the “phonon” concept from solid-state physics to refer to the “coherence wave” (the off-diagonal term of the density matrix of molecular motion) in molecular gases was introduced by Russell and co-workers.^42,66,102,103^ The use of “phonon” to refer to the coherence wave also minimizes potential confusion in discussions about the “coherent” generation of a Raman process, which pertains to interactions involving temporal phases between pump and Raman pulses. Details of the generalization and justification are presented in Sec. 2 of the supplementary material.

To establish a complete physical picture of SRS, it will be valuable to develop a unified theory that applies across temporal regimes. Despite the long history of Raman scattering and its widespread use in both the scientific and industrial communities, such a theory is still lacking. As an example of where it will be useful, we highlight techniques for ultrashort Stokes-pulse generation based on the interactions of multiple pulses with phonons.^53,86–90^ In this process, it is essential for the pump pulse duration to be long enough to prevent distortions and avoid competing nonlinear processes. Only with the unified theory can the interaction of impulsively excited phonons and a transient Raman process be fully understood. As another example, most prior theoretical works on Raman scattering neglect other effects that can play critical roles.^102,104–110^ A delicate balance between phonon annihilation (anti-Stokes processes) and creation (Stokes processes) can occur through wave-vector matching of the interaction of Stokes, pump, and anti-Stokes waves. In this situation, no new phonons are generated, resulting in no new photons as well. This phenomenon is referred to as “Raman gain suppression,” where both wave-vector matching and Kerr-induced four-wave mixing (FWM) play a non-negligible role. While its impact has been thoroughly explored in the steady-state regime,^85,111–114^ it has only been examined in the vicinity of perfect wave-vector matching in the transient regime.^115^ Even recent investigations into transient Raman gain suppression in HCFs rely on the steady-state gain model,^90,102,103,110^ which necessitates an explanation for its effectiveness. Unlike the case of steady-state Raman gain with FWM, a straightforward analytical expression for transient Raman gain with FWM is currently lacking.

In addition to the absence of a theory that can address multiple temporal regimes, there is a deficiency in our understanding of vector (i.e., polarization) effects in SRS. This knowledge gap can lead to uncontrolled physical phenomena, as exemplified by the depolarization observed during the SSFS process in a H_2_-filled anti-resonant fiber.^69^ Almost all prior studies employ scalar models. Vector aspects have been addressed within the steady-state formalism,^84,116–121^ but a model that can elucidate the temporal dynamics of vector Raman interactions does not exist. Typical Raman-scattering mechanisms involve transitions between vibrational and rotational states. Vibrational SRS in solids and liquids has been the focus of many prior studies, and its vector aspects in crystalline materials have been addressed extensively.^122–126^ However, vibrational SRS is isotropic in gaseous environments. On the other hand, rotational SRS exhibits pronounced anisotropy owing to its inherent connection with exchange of angular momentum, and, as a result, exhibits distinct responses to light with varying polarizations. Only recently has a tensor formulation been introduced to simulate rotational SRS in air to mitigate laser-plasma instabilities through nonlinear spectral broadening with elliptically polarized light.^127^ General aspects of vector SRS interactions are still not fully understood.

This *Perspective* aims to address these gaps in our current understanding of SRS, with a focus on processes relevant to Raman generation with picosecond- or femtosecond-duration pulses in gas-filled hollow waveguides. Although steady-state Raman gain has been investigated extensively, transient Raman gain and its associated dynamics have not been thoroughly examined. Previous studies have focused on Raman effects influenced by electronic-induced nonlinear phase modulations.^128–130^ However, the potentially significant contribution of the Raman response to nonlinear phases has been overlooked. Although nonlocal interactions through phonon waves have been extensively investigated,^91–95,101,131–143^ a complete picture only emerges through analysis that can handle different temporal regimes. Finally, the vector Raman response of gases has largely been neglected.

Here, we present a theoretical model of pulse propagation in gas-filled HCFs that covers all temporal regimes (steady-state, transient, and impulsive) of SRS as well as vector effects. Application of the theory to previous experiments will serve as a partial review of recent activities in the area and set the context for future directions. The theory aids conceptual and intuitive understanding of previously observed phenomena, in some cases from new points of view, and makes predictions about processes that are currently under investigation, or will be in the future. The results also illustrate new approaches to controlling Raman scattering for wavelength-conversion applications. Analytic expressions provide qualitative and quantitative descriptions of example phenomena, and detailed numerical simulations with representative experimental parameters back up and complement the analytical results. Although the presented model is capable of treating spontaneous Raman scattering (and all numerical results presented in this Perspective were calculated with the inclusion of spontaneous Raman scattering), the quantum nature of the initiation process does not play a key role in the processes examined here, which are in the nonlinear regime of Raman scattering (i.e., when the gain is high and saturated). Thus, we do not discuss the initiation process. For treatments of spontaneous processes and the initiation of Raman scattering, readers are referred to excellent prior works.^13,107,144–149^

The rest of this Perspective is organized as follows. Sec. II presents the nonlinear pulse propagation equation, which is the foundation of the unified theory, numerical simulations, and subsequent analysis presented in this Perspective. Section III applies the scalar model to phenomena in all three temporal regimes, with emphases on the behavior of the Raman gain and the interplay of Raman and electronic nonlinearities that govern a variety of ultrafast phenomena. In Sec. IV, the vector Raman response is derived and applied to polarization effects in vibrational and rotational SRS in diatomic molecules. Section V offers some perspectives on possible future directions for the field of SRS physics in gas-filled HCF.

The computational process is described in Secs. 11–13 of the supplementary material, along with some technical issues that are encountered in simulations of SRS in gases. The numerical code used to produce all of the simulation results presented in this Perspective is implemented in the MATLAB computing platform and is freely available.

## II. PULSE PROPAGATION EQUATION

Raman scattering is generally analyzed with one of the two models. One model is based on the assumption of a harmonic oscillator for the excited vibrational mode,^85,139–141^ while the other treats the density matrix for the vibrational or rotational states.^68,73,90,101,103,105,107–110,115,142,150–153^ Although they are not discussed in this Perspective, those models underlie our theoretical approach. Readers are referred to Sec. 3 of the supplementary material for detailed descriptions and comparisons of the two models.

The unidirectional pulse propagation equation (UPPE) has been commonly used to study nonlinear wave propagation in gas-filled HCF due to its ability to handle broadband phenomena and few-cycle dynamics.^154^ The starting point of this work is the vector UPPE [Eq. (1)],^154–157^ whose derivation is provided in Sec. 4 of the supplementary material.In the basis of linear polarizations,

$$\begin{array}{ll} \partial_{z}A_{x}\left(z,\Omega \right) & =i\left[\beta_{x}\left(\omega \right)-\left(\beta_{\left(0\right)}+\beta_{\left(1\right)}\Omega \right)\right]A_{x}\left(z,\Omega \right)+i\omega \kappa \left\{\kappa_{e}F\left[\left(|A_{x}{|}^{2}+\frac{2}{3}|A_{y}{|}^{2}\right)A_{x}+\frac{1}{3}A_{y}^{2}A_{x}^{*}\right]\right.\\ & \;+\left.F\left[\left[\left(\frac{1}{2}R_{a}\right)*\left(|A_{x}{|}^{2}+|A_{y}{|}^{2}\right)\right]A_{x}+\left[\left(\frac{1}{2}R_{b}\right)*|A_{x}{|}^{2}\right]A_{x}+\left[\left(\frac{1}{4}R_{b}\right)*\left(A_{x}A_{y}^{*}+A_{x}^{*}A_{y}\right)\right]A_{y}\right]\right.\\ & \;+\left.F\left[\left[2\text{Re}\left[\frac{1}{2}\left({\tilde{\Gamma }}_{xx}^{R_{a}}+{\tilde{\Gamma }}_{yy}^{R_{a}}\right)+\frac{1}{2}{\tilde{\Gamma }}_{xx}^{R_{b}}\right]\right]A_{x}+\left[\frac{1}{4}\left[{\tilde{\Gamma }}_{xy}^{R_{b}}+{\left({\tilde{\Gamma }}_{xy}^{R_{b}}\right)}^{*}\right]\right]A_{y}\right]\right\}, \end{array}$$ (1a)

with an analogous equation for $\partial_{z}A_{y}\left(z,\Omega \right)$.

In the basis of circular polarizations,

$$\begin{array}{ll} \partial_{z}A_{+}\left(z,\Omega \right) & =i\left[\beta_{+}\left(\omega \right)-\left(\beta_{\left(0\right)}+\beta_{\left(1\right)}\Omega \right)\right]A_{+}\left(z,\Omega \right)+i\omega \kappa \left\{\kappa_{e}F\left[\left(\frac{2}{3}|A_{+}{|}^{2}+\frac{4}{3}|A_{-}{|}^{2}\right)A_{+}\right]\right.\\ & \;+\left.F\left[\left[\left(\frac{1}{2}R_{a}+\frac{1}{4}R_{b}\right)*\left(|A_{+}{|}^{2}+|A_{-}{|}^{2}\right)\right]A_{+}+\left[\left(\frac{1}{2}R_{b}\right)*\left(A_{+}A_{-}^{*}\right)\right]A_{-}\right]\right.\\ & \;+\left.F\left[\left[2\text{Re}\left[\frac{1}{2}\left({\tilde{\Gamma }}_{++}^{R_{a}}+{\tilde{\Gamma }}_{--}^{R_{a}}\right)+\frac{1}{4}\left({\tilde{\Gamma }}_{++}^{R_{b}}+{\tilde{\Gamma }}_{--}^{R_{b}}\right)\right]\right]A_{+}+\left(\frac{1}{2}{\tilde{\Gamma }}_{+-}^{R_{b}}\right)A_{-}\right]\right\}, \end{array}$$ (1b)

with an analogous equation for $\partial_{z}A_{-}\left(z,\Omega \right)$.

The equations [Eq. (1)] include dispersion as well as instantaneous electronic and delayed Raman contributions to the nonlinearity. *A*_*p*_(*z*, *T*) is the envelope of the electric field (in $\sqrt{W}$) of polarization mode *p*, whose Fourier transform is $A_{p}\left(z,\Omega \right)=F\left[A_{p}\left(z,T\right)\right]$. The Fourier transform is applied with respect to angular frequency Ω = *ω* − *ω*_0_, where *ω*_0_ is the center angular frequency of the numerical frequency window required to cover the investigated physical phenomena. *β*_*p*_ is the propagation constant of mode *p*, obtained from the dispersion formula either for anti-resonant fiber^158^ or for capillary;^159^ *β*_(0)_ and *β*_(1)_ are to reduce the propagating global-phase increment to facilitate simulations in which *β*_(1)_ is the inverse group velocity of the moving reference frame that introduces the delayed time *T* = *t* − *β*_(1)_*z*. $\kappa \left(\omega \right)=1/\left(\varepsilon_{0}^{2}{\left[n_{\text{eff}}\left(\omega \right)\right]}^{2}c^{2}A_{\text{eff}}\left(\omega \right)\right)$ and $\kappa_{e}\left(\omega \right)=\left(3/4\right)\varepsilon_{0}\chi_{\text{electronic}}^{\left(3\right)}\left(\omega \right)$. $A_{\text{eff}}\left(\omega \right)=1/\left(\int {\left|F\right|}^{4}d^{2}x\right)$ is the effective mode field area, and *F*(*x*, *y*) is the normalized mode profile with ∫|*F*|^2^d*x*d*y* = 1. $\chi_{\text{electronic}}^{\left(3\right)}\left(\omega \right)$ is the third-order nonlinear susceptibility of the electronic response (in m^2^/V^2^) that is proportional to the gas number density; $R_{a}\left(t\right)$ and $R_{b}\left(t\right)$ are isotropic and anisotropic Raman response functions that are also proportional to the gas number density and will be derived later [Eq. (31)]. They result from the polarization^160^

$$\vec{P}\left(t\right)=\int_{-\infty }^{\infty }\chi^{\left(3\right)}\;\vdots \;\vec{E}\left(t-t_{1}\right)\vec{E}\left(t-t_{2}\right)\vec{E}\left(t-t_{3}\right)\;dt_{1}\;dt_{2}\;dt_{3},$$ (2)

where

$$\chi^{\left(3\right)}\left(t_{1},t_{2},t_{3}\right)=\delta \left(t_{1}\right)\delta \left(t_{2}-t_{3}\right){\hat{R}}^{\mathrm{ijk\ell}}\left(t_{3}\right),$$ (3a)

$$\begin{array}{ll} {\hat{R}}^{\mathrm{ijk\ell}}\left(t\right) & =\varepsilon_{0}\chi_{\text{electronic}}^{\left(3\right)}\frac{\delta^{ij}\delta^{k\ell }+\delta^{ik}\delta^{j\ell }+\delta^{i\ell }\delta^{jk}}{3}\delta \left(t\right)\\ & \;+R_{a}\left(t\right)\delta^{ij}\delta^{k\ell }+R_{b}\left(t\right)\frac{\delta^{ik}\delta^{j\ell }+\delta^{i\ell }\delta^{jk}}{2}. \end{array}$$ (3b)

The supplementary material of the present article includes a detailed derivation of the UPPE for a multimode system, which includes both transverse modes and polarization modes, along with both isotropic and anisotropic Raman responses. For the sake of simplicity in illustrating the underlying physics, this Perspective considers only *a single transverse mode* [Eq. (1)]. The conclusions drawn here can be extended to model nonlinear dynamics involving multiple transverse modes^42,78–82,103^ using the multimode UPPE in the supplementary material.

The quantum nature of Raman scattering is treated by incorporating the quantum-statistical Langevin terms ${\tilde{\Gamma }}_{mn}^{R_{r}}\left(z,T\right)$ (*r* = *a* or *b*), which correspond to vacuum fluctuations and collisional dephasing. They obey the spectral correlation given by^144,148,149,161,162^

$$\begin{array}{ll} \left\langle {\tilde{\Gamma }}_{mn}^{R_{r}}\left(z,\Omega \right){\left({\tilde{\Gamma }}_{mn}^{R_{r}}\left(z^{\prime },\Omega^{\prime }\right)\right)}^{*}\right\rangle & =2\frac{2\hbar }{C_{F}\kappa S_{mn}^{R_{r}}}\left|F,\left[R_{r}\right],\left(\Omega \right)\right|\left[n_{\text{th}}\left(\left|\Omega \right|\right)\right.\\ & \;\left.+\Theta \left(-\Omega \right)\right]\delta \left(z-z^{\prime }\right)\delta \left(\Omega -\Omega^{\prime }\right), \end{array}$$ (4)

where Ω′ = *ω*′ − *ω*_0_. The factors resulting from overlap integrals $S_{xx}^{R_{a}}=S_{yy}^{R_{a}}=S_{xx}^{R_{b}}=S_{++}^{R_{a}}=S_{--}^{R_{a}}=S_{+-}^{R_{b}}=1$ and $S_{xy}^{R_{b}}=S_{++}^{R_{b}}=S_{--}^{R_{b}}=1/2$. $C_{F}$ is the constant of Fourier Transform where $F\left[f\right]\left(\omega \right)=C_{F}\int_{-\infty }^{\infty }f\left(t\right)e^{i\omega t}dt$ (see Sec. 1 of the supplementary material for the tutorial regarding conventions and symbols of Fourier transform). The thermal phonons obey the Bose–Einstein distribution $n_{\text{th}}\left(\left|\Omega \right|\right)={\left[e^{\hbar |\Omega |/k_{B}T}-1\right]}^{-1}$. Θ(−Ω) is the Heaviside step function. The Langevin functions ${\tilde{\Gamma }}_{mn}^{R_{r}}$’s for different vector contributions represent different random values from the same correlation relation of the Raman response function $R_{r}$. Section 4 of the supplementary material describes the numerical implementation and the symmetry requirement of Langevin terms. These Langevin terms account for spontaneous Raman scattering. Spontaneous Stokes emission results from the property that Θ(−Ω) ≠ 0 for Ω < 0, representing vacuum fluctuations of phonons. On the other hand, spontaneous anti-Stokes emission is weak unless the thermal phonon population is appreciable. Raman scattering can be described as a parametric process that involves phonons and photons.^163^ This was employed by von Foerster and Glauber to describe the phonon evolution^164^ and later used to unify spontaneous and stimulated Raman scattering within a single Maxwell–Bloch theoretical framework by Raymer and Mostowski.^107,145^ This framework assumes a heavily populated ground state, which aligns with the perturbative Raman regime that leads to the derived Raman response functions later in Eq. (31). A new formulation is required if the perturbative assumption is relaxed and is beyond the scope of this Perspective.^165^ Our UPPE formulation with Langevin terms is the generalization of the quantum-electrodynamical framework of Raymer and Mostowski to the waveguide environment.^144,148,149^ Equation (4) is the c-number equation that corresponds to the operator’s commutator relation. Although the equations above should be able to simulate quantum phenomena, in this Perspective, we focus on the nonlinear or highly pump-depleted regime, where quantum fluctuations from spontaneous Raman scattering are minimized. In the linear Raman regime, Stokes photons, scattered by spontaneous emission, exhibit ∼100% energy fluctuations with a negative-exponential statistical distribution.^166,167^ However, in the nonlinear regime, the output field is stabilized, with a distribution that is statistically peaked at the average value.^168^ Thus, in this Perspective, “Raman generation” predominantly denotes the manifestation of SRS, that is, Raman amplification, rather than spontaneous Raman scattering. Spontaneous Raman scattering does contribute to the fluctuations observed in the results represented in Figs. 4 and 10.

Shot noise of the input field is modeled semi-classically by including one noise photon per spectral discretization bin.^162,169–171^ Section 4 of the supplementary material includes more details about its numerical implementation. It is worth mentioning that there are other input-pulse noise models, obtained by either carefully modeling the buildup of lasing of the source or adding a Lorentzian noise spectrum derived from a phase-diffusion model.^170^ However, they aim to realistically model the input-pulse noise spectrum. Gases usually have large Raman transition frequencies, and such relatively narrowband noise spectra have insignificant impact on nonlinear processes. Thus, more-realistic noise models are unnecessary for the purposes of this work.

Two types of hollow-core fibers are considered below. In HCF, the dispersion has components of anomalous waveguide dispersion and normal gas dispersion.^159^ An anti-resonant fiber with a small 30-*μ*m core diameter (and 300-nm tube-wall thickness) is assumed for analysis of processes with anomalous dispersion, while a Ag-coated capillary with a large 300-*μ*m core diameter is assumed for those with normal dispersion.

Numerical simulations assume that the HCFs are filled with H_2_, N_2_, or O_2_. These gases are currently the most prevalent Raman-active gases in use. Numerical values of their parameters, as well as initial validation of our model, are provided in Secs. 11 and 12 of the supplementary material.

## III. SCALAR RAMAN RESPONSE WITH FWM

In this section, we aim to present Raman effects across different regimes using a unified model based on a scalar UPPE. Several known phenomena will be reexamined with this unified framework. It covers not only Raman responses to Stokes generation but also coupling with anti-Stokes waves arising from both electronic- and Raman-induced FWM. FWM underlies Raman gain suppression, Raman-induced phase modulations, and other related effects, which will be discussed later.

Raman gain is a fundamental quantity in SRS physics, so Raman gain suppression is a critical concept. Since the groundbreaking research conducted by Bloembergen and Shen on Raman gain suppression,^111^ there has been a significant focus on understanding parametric effects on Raman gain.^112–114,116–118,120,121,172–177^ The small dispersion of gases translates into small wave-vector mismatch among Stokes, pump, and anti-Stokes waves, so the effect of Raman gain suppression (which is the strongest at perfect wave-vector matching) in gas-filled HCF cannot be ignored. Argon has been added to hydrogen to modify the overall dispersion profile, creating wave-vector mismatch and mitigating gain suppression,^110^ for example. Higher-order transverse modes have been introduced to excite intermodal coherence waves to disrupt the Stokes/anti-Stokes balance and allow the Stokes wave to grow.^42^ In this paper, we focus mostly on the Raman gain of Stokes waves; for anti-Stokes evolution, please see Sec. 8 of the supplementary material for details.

To study Raman gain and other SRS effects, we start from the scalar UPPE [*A*_*y*_ = 0, along with corresponding Langevin terms ${\tilde{\Gamma }}_{yy}^{R_{a}}={\tilde{\Gamma }}_{xy}^{R_{b}}=0$, in Eq. (1a)],

$$\begin{array}{ll} \partial_{z}A\left(z,\Omega \right) & =i\left[\beta \left(\omega \right)-\left(\beta_{\left(0\right)}+\beta_{\left(1\right)}\Omega \right)\right]A\left(z,\Omega \right)\\ & \;+i\omega \kappa \left\{\kappa_{e}F\left[|A{|}^{2}A\right]+F\left[A\left(R*|A{|}^{2}+2\Gamma \right)\right]\right\}, \end{array}$$ (5)

where $R=\left(R_{a}+R_{b}\right)/2$ and $\Gamma =\text{Re}\left[{\tilde{\Gamma }}_{xx}^{R_{a}}+{\tilde{\Gamma }}_{xx}^{R_{b}}\right]/2$. To simplify the analysis, a one-component Raman response is considered: $R\left(t\right)=\Theta \left(t\right)R^{\text{coeff}}e^{-\gamma_{2}t}\;\sin\left(\omega_{R}t\right)$, where the dephasing rate *γ*_2_ = 1/*T*_2_. (Simulation results shown below include both vibrational and rotational SRS.) If the field is composed of Stokes (*ω*^*S*^ = *ω*^*P*^ − △*ω*), pump (*ω*^*P*^), and anti-Stokes (*ω*^*AS*^ = *ω*^*P*^ + △*ω*) waves,

$$\begin{array}{ll} A\left(z,t\right) & =A^{P}\left(z,t\right)e^{i\left(\beta^{P}z-\omega^{P}t\right)}+A^{S}\left(z,t\right)e^{i\left(\beta^{S}z-\omega^{S}t\right)}\\ & \;+A^{AS}\left(z,t\right)e^{i\left(\beta^{AS}z-\omega^{AS}t\right)}. \end{array}$$ (6)

With *β*_(0)_ = *β*_(1)_ = 0 for analysis purposes, we obtain from Eq. (5)

$$\partial_{z}A^{P}\left(z,t\right)=i\omega^{P}\kappa^{P}\left[\kappa_{e}^{P}{\left|A^{P}\right|}^{2}A^{P}+R_{1}A^{P}\right],$$ (7a)

$$\begin{array}{ll} \partial_{z}A^{S}\left(z,t\right) & =i\omega^{S}\kappa^{S}\left\{2\kappa_{e}^{S}{\left|A^{P}\right|}^{2}A^{S}+e^{i△\beta z}\kappa_{e}^{S}{\left(A^{P}\right)}^{2}{\left(A^{AS}\right)}^{*}\right.\\ & \left.\;+R_{1}A^{S}+R_{2;P,P^{*},S}+e^{i△\beta z}R_{2;P,P,AS^{*}}\right.\\ & \left.\;+\left[\Gamma^{\left(\Omega <0\right)}+e^{i△\beta z}{\left(\Gamma^{\left(\Omega >0\right)}\right)}^{*}\right]A^{P}\right\}, \end{array}$$ (7b)

$$\begin{array}{ll} \partial_{z}{\left(A^{AS}\left(z,t\right)\right)}^{*} & =-i\omega^{AS}\kappa^{AS}\left\{2\kappa_{e}^{AS}{\left|A^{P}\right|}^{2}{\left(A^{AS}\right)}^{*}\right.\\ & \;\left.+e^{-i△\beta z}\kappa_{e}^{AS}{\left[{\left(A^{P}\right)}^{*}\right]}^{2}A^{S}+R_{1}{\left(A^{AS}\right)}^{*}\right.\\ & \;\left.+R_{2;P^{*},P,AS^{*}}+e^{-i△\beta z}R_{2;P^{*},P^{*},S}\right.\\ & \;\left.+\left[{\left(\Gamma^{\left(\Omega >0\right)}\right)}^{*}+e^{-i△\beta z}\Gamma^{\left(\Omega <0\right)}\right]{\left(A^{P}\right)}^{*}\right\}, \end{array}$$ (7c)

where weak Stokes/anti-Stokes waves are assumed so that pump depletion is negligible. The Langevin term, before taking only its real part, consists of positive- and negative-frequency parts: $\Gamma \left(t\right)=\text{Re}\left[\Gamma^{\left(\Omega <0\right)}e^{i\left(-△\beta^{S}z+△\omega t\right)}+\Gamma^{\left(\Omega >0\right)}e^{i\left(-△\beta^{AS}z-△\omega t\right)}\right]$, where △*β*^*i*^ = *β*^*P*^ − *β*^*i*^ (*i* = *S* or *AS*). △*β* = 2*β*^*P*^ − *β*^*S*^ − *β*^*AS*^ is responsible for wave-vector matching among the three waves. The Raman integrals are

$$R_{1}=R*{\left|A^{P}\right|}^{2},$$ (8a)

$$R_{2;i,j,k}=A^{i}\int_{-\infty }^{t}R\left(t-\tau \right)A^{j}\left(\tau \right)A^{k}\left(\tau \right)e^{-i△\omega \left(t-\tau \right)}\;d\tau .$$ (8b)

In gases, the Raman response is narrowband, and frequencies detuned from *ω*_*R*_ do not play an important role. We assume △*ω* = *ω*_*R*_ to simplify the subsequent analysis. The integral $R_{1}$ is responsible for the Raman-induced index change (which underlies phase modulations), and $R_{2;i,j,k}$ is usually responsible for the Raman gain, where i*, j*, and k* represent the complex conjugates of the corresponding fields. Equations (7) and (8) are the basis of the analysis of distinct temporal regimes below.

### A. Steady-state regime (△*t*_*p*_ ≫ *T*_2_)

We start with the steady-state regime. While this regime has been extensively explored, it serves as a reference point for subsequent discussions on the less-explored transient and impulsive regimes. Additionally, most prior studies ignore the electronic response.^85,102,103,105,110^ The few studies that include the electronic nonlinearity treat silica fiber, where SRS is weak.^172,173,178^ A thorough investigation of strong Raman response plus electronic nonlinearity in gases is still lacking.

In this regime, phonons decay on a time scale much shorter than the pulse duration, so the phonon waves are affected only by the instantaneous temporal segment of the pulse. Therefore, the Raman integrals [Eq. (8)] can be approximated as

$$R_{1}\left(z,t\right)\approx I_{R}{\left|A^{P}\left(t\right)\right|}^{2}\approx R^{\text{coeff}}\frac{{\left|A^{P}\left(t\right)\right|}^{2}}{\omega_{R}},$$ (9a)

$$\begin{array}{ll} R_{2;i,j,k}\left(z,t\right) & \approx R_{\text{ss}}A^{i}\left(z,t\right)A^{j}\left(z,t\right)A^{k}\left(z,t\right)\\ & \approx \left[R^{\text{coeff}}\left(-\frac{i}{2}T_{2}+\frac{1}{4\omega_{R}}\right)\right]A^{i}A^{j}A^{k}, \end{array}$$ (9b)

where $I_{R}=\int_{-\infty }^{t}R\left(t-\tau \right)\;d\tau =R^{\text{coeff}}\frac{\omega_{R}}{\omega_{R}^{2}+\gamma_{2}^{2}}$ and $R_{\text{ss}}=\int_{-\infty }^{t}R\left(t-\tau \right)e^{-i\omega_{R}\left(t-\tau \right)}\;\mathrm{d\tau}=R^{\text{coeff}}\frac{\omega_{R}}{2i\omega_{R}\gamma_{2}+\gamma_{2}^{2}}$ is the Fourier transform of *R*(*t*) at −*ω*. *A*^*i*^*A*^*j*^*A*^*k*^ in $R_{2;i,j,k}$ is either ${\left|A^{P}\right|}^{2}A^{k}$ or ${\left|A^{P}\right|}^{2}A^{k}e^{2i\phi^{P}\left(z,t\right)}$, where *ϕ*^*P*^(*z*, *t*) is the phase of *A*^*P*^. Further approximations in Eq. (9) are made by taking *ω*_*R*_ ≫ *γ*_2_, which is typically the case in gases at pressures less than 100 bar. Since $R_{1}\propto {\left|A^{P}\right|}^{2}$, the Raman-induced index change leads to an instantaneous Kerr effect. With these integrals and assuming that *κ*^*S*^ ≈ *κ*^*AS*^ ≈ *κ* and $\kappa_{e}^{S}\approx \kappa_{e}^{AS}\approx \kappa_{e}$, the Raman amplitude-gain with FWM [the quantity *g* in *A*^*S*^ ∼ e^*gz*^*A*^*S*^*(z=0*)] becomes (see Sec. 8 of the supplementary material for the derivation details)

$$\begin{array}{ll} g_{\text{ss}} & =\kappa \omega_{R}\;Im\left[R_{\text{ss}}\right]{\left|A^{P}\right|}^{2}\\ & \;+\left|\text{Re},\left[\frac{1}{2}\sqrt{2\kappa \left[\left(\sqrt{\omega^{S}\omega^{AS}}+\omega^{P}\right)\left(\kappa_{e}+R_{\text{ss}}\right)\right]{\left|A^{P}\right|}^{2}-△\beta }\right.\right.\\ & \;\left.\times ,\left.\sqrt{2\kappa \left[\left(\sqrt{\omega^{S}\omega^{AS}}-\omega^{P}\right)\left(\kappa_{e}+R_{\text{ss}}\right)\right]{\left|A^{P}\right|}^{2}+△\beta }\right]\right|. \end{array}$$ (10)

To gain insights into Eq. (10), the Raman shift is assumed to be small (*ω*_*R*_ ≪ *ω*^*P*^), and Eq. (10) can then be reduced to

$$\begin{array}{ll} g_{\text{ss}} & \approx \kappa \omega_{R}\;Im\left[R_{\text{ss}}\right]{\left|A^{P}\right|}^{2}\\ & \;+\left|\text{Re},\left[\frac{1}{2}\sqrt{\left[4\kappa \omega^{P}\left(\kappa_{e}+R_{\text{ss}}\right){\left|A^{P}\right|}^{2}-△\beta \right]△\beta }\right]\right|. \end{array}$$ (11)

**FIG. 2.**
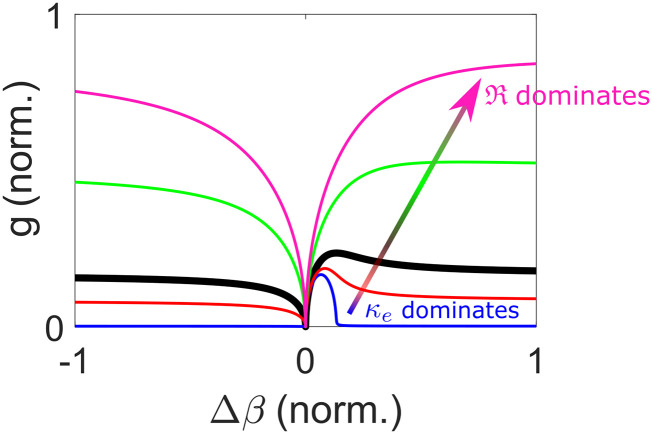
Steady-state Raman gain *g* [Eq. (11)] with respect to wave-vector mismatch △*β*. The arrow indicates the trend of increasing ImRss (blue → red → black → green → pink) with fixed $\text{Re}\left[\kappa_{e}+R_{\text{ss}}\right]$. The thick black line is the line with equal magnitudes of real and imaginary parts of $\left(\kappa_{e}+R_{\text{ss}}\right)$.

Since the gain increases monotonically with ${\left|A^{P}\right|}^{2}$, the Raman generation depends on the instantaneous intensity of the pump and, hence, depletes only the pulse center. The second △*β* term under the square-root sign in Eq. (11) corresponds to Raman gain suppression (minimum *g*_ss_ at △*β* = 0), while the first term in the square brackets corresponds to modulation instability. The latter creates a gain peak when the Raman response is weak (ImRss≪Reκe+Rss; blue, red, and black lines in Fig. 2).^172,173,179^ Such a gain shape has been widely studied in media with negligible Raman nonlinearity, creating the conventional Kerr-induced FWM gain, where both the Stokes and anti-Stokes waves are maximally generated through the parametric process at $∆\beta \approx 2\kappa \omega^{P}\kappa_{e}{\left|A^{P}\right|}^{2}$.^180^ However, in Raman-active gases, due to the slow dephasing, ImRss (∝*T*_2_ in gases) can be a few thousand times larger than $\left|\text{Re}\left[\kappa_{e}+R_{\text{ss}}\right]\right|$ (in 20-bar H_2_, it is about 7 000 times for the Q(1) SRS). This results in an almost-monotonically increasing gain away from △*β* = 0 until it asymptotically reaches the value of Raman gain without FWM, κωSImRssAP2 (green and pink lines in Fig. 2), which is consistent with the prior works with negligible electronic nonlinearity.^85,102^

This section analytically calculates the steady-state Raman integrals [Eq. (9)] and Raman gain [Eq. (10)], incorporating FWM arising from electronic and Raman nonlinearities. The variation of Raman gain with the strength of the electronic and Raman responses is illustrated, ranging from negligible Raman response to the pronounced response in Raman-active gases (Fig. 2).

### B. Transient regime (△*t*_*p*_ ≪ *T*_2_, △*t*_*p*_ ≫ *T*_*R*_)

Because gases typically possess dephasing times greater than ∼100 ps at moderate gas pressures, experiments performed with picosecond or femtosecond pulses are commonly in the transient Raman regime. Additionally, gases usually exhibit large rotational and vibrational Raman transition frequencies. Consequently, the condition of having a pulse duration longer than the phonon oscillation period is frequently met.

The biggest challenge in analyzing transient SRS is the calculation of the Raman integrals, $R_{1}$ and $R_{2;i,j,k}$ [Eq. (8)]. During pulse propagation, *A*^*P*^ acquires a *z*-dependent phase through self-phase modulations (SPM), while *A*^*i*^ (*i* = *S* or *AS*) acquires it through cross-phase modulations (XPM) from the pump. These phase modulations are intensity- and, thus, time-dependent, which leave the Raman integrals analytically unsolvable. To simplify the problem, we assume that all three pulses have an identical flat-top temporal structure, leading to time-independent phase modulations within the pulse. In addition, it is well-known that, when the gain is high and saturated, both the Stokes and anti-Stokes signals exhibit the same temporal phase structure as the pump.^105^ This results from amplifying only those frequencies that can maximize the Raman integrals, which always include either $A^{P}{\left(A^{k}\right)}^{*}$ or ${\left(A^{P}\right)}^{*}A^{k}$ [Eq. (7)]. As a result, *A*^*i*^ = *C*^*i*^*A*^*P*^ (*i* = *S* or *AS*, and |*C*^*i*^| ≪ 1 with *C*^*i*^ being real-valued) is reasonably assumed. Furthermore, the flat-top assumption facilitates the analysis of temporal characteristics of the Raman processes. While we make the “flat-top” assumption here, the validity of the following discussions extends more generally to pulses with negligible nonlinear phase modulations in comparison to Raman responses.

In this regime, because phonons decay slowly, we assume *γ*_2_ ≈ 0. To solve the Raman integrals [Eq. (8)], we introduce the following mathematical approximations for an arbitrary smooth real-valued function *f*(*t*) when △*t*_*p*_ ≫ *T*_*R*_:

$$\int_{-\infty }^{t}\left[\sin\left(\omega_{R}\left(t-\tau \right)\right)\right]f\left(\tau \right)\;d\tau \approx \frac{f\left(t\right)}{\omega_{R}},$$ (12a)

$$\int_{-\infty }^{t}\left[\sin\left(\omega_{R}\left(t-\tau \right)\right)\right]f\left(\tau \right)e^{-i\omega_{R}\left(t-\tau \right)}\;d\tau \approx -\frac{i}{2}\int_{-\infty }^{t}f\left(\tau \right)\;d\tau +\frac{f\left(t\right)}{4\omega_{R}}.$$ (12b)

These approximations are derived by partitioning one into multiple integrals, each with a small interval equal to *T*_*R*_. Since *T*_*R*_ ≪ △*t*_*p*_, *f*(*τ*) remains nearly stationary within each interval [*t*_*n*+1_, *t*_*n*_], allowing us to approximate it as $f\left(t_{n+1}\right)+f^{\prime }\left(t_{n+1}\right)\left(\tau -t_{n+1}\right)$, which leads to the final results [Eq. (12)]. Details are in Sec. 7 of the supplementary material.

With these approximations [Eq. (12)], the Raman integrals in the transient regime are approximated as

$$R_{1}\left(z,t\right)\approx R^{\text{coeff}}\frac{{\left|A^{P}\left(t\right)\right|}^{2}}{\omega_{R}},$$ (13a)

$$\begin{array}{ll} R_{2;i,j,k}\left(z,t\right) & \approx R_{\text{tr}}A^{i}C^{k}\\ & =\left[R^{\text{coeff}}\left(-\frac{i}{2}\int_{-\infty }^{t}{\left|A^{P}\left(\tau \right)\right|}^{2}\;d\tau +\frac{{\left|A^{P}\left(t\right)\right|}^{2}}{4\omega_{R}}\right)\right]A^{i}C^{k}, \end{array}$$ (13b)

where *A*^*P*^*C*^*k*^ = *A*^*k*^ and $A^{P}{\left(C^{k}\right)}^{*}={\left(A^{k}\right)}^{*}e^{2i\phi^{P}\left(z,t\right)}$. These equations are similar to Eq. (9), with the only difference lying in the imaginary part where ${\left|A^{P}\right|}^{2}T_{2}$ in the steady-state regime corresponds to $\int_{-\infty }^{t}{\left|A^{P}\left(\tau \right)\right|}^{2}\;\;d\tau$ in the transient regime. The similarity permits the process applied in the steady-state regime to be applied to obtain an analytic expression for the Raman gain in the transient regime,

$$\begin{array}{ll} g_{\text{tr}} & =\kappa \omega_{R}\;Im\left[R_{\text{tr}}\right]\\ & \;+\left|\text{Re},\left[\frac{1}{2}\sqrt{2\kappa \left[\left(\sqrt{\omega^{S}\omega^{AS}}+\omega^{P}\right)\left(\kappa_{e}{\left|A^{P}\right|}^{2}+R_{\text{tr}}\right)\right]-△\beta }\right.\right.\\ & \;\times \left.\left.\sqrt{2\kappa \left[\left(\sqrt{\omega^{S}\omega^{AS}}-\omega^{P}\right)\left(\kappa_{e}{\left|A^{P}\right|}^{2}+R_{\text{tr}}\right)\right]+△\beta }\right]\right|. \end{array}$$ (14)

The similarity of the transient equations [Eqs. (13) and (14)] to the steady-state equations [Eqs. (9) and (10)] allows several phenomena in the steady-state regime to be transferred to the transient regime, such as the Raman gain shape and the resulting Raman gain suppression at △*β* = 0. This explains why the expression for the steady-state Raman gain has proven useful in predicting the qualitative features of transient Raman gain suppression.^42,90,102,103,110^

**FIG. 3.**
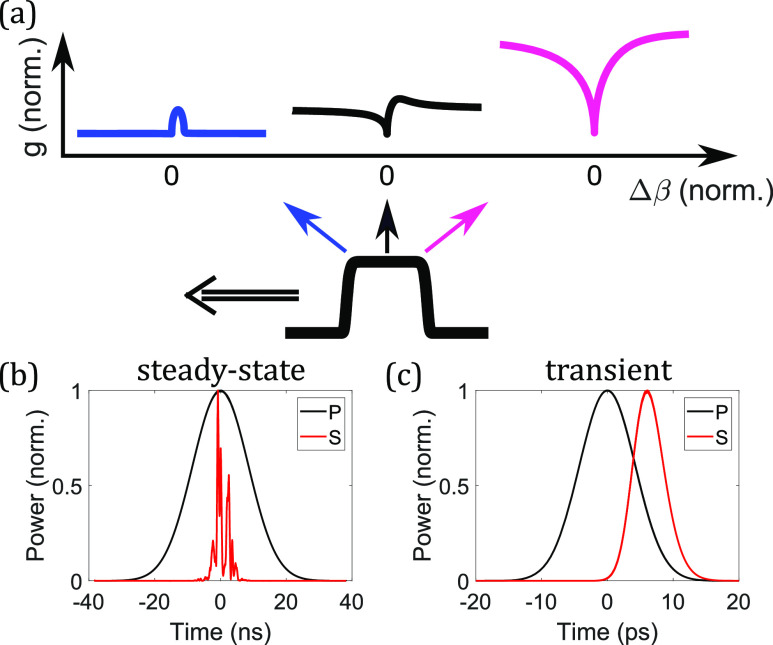
(a) Varying Raman gain (extracted from Fig. 2) at different positions of a pulse in the transient Raman regime. Left represents the leading edge. Temporal profiles of the pump (P) and the Stokes (S) waves from the rotational Raman-Stokes generation in (b) the steady-state and (c) the transient regimes are shown. (b) A 20-ns and 70-*μ*J and (c) a 10-ps and 1-*μ*J Gaussian pulses at 1030 nm are launched into a 1-m-long anti-resonant fiber, filled with 30-bar H_2_. At this pressure, the dephasing time is 100 ps. Despite the linearly polarized fields, the dominant process is S(1) rotational SRS^181^ due to the stronger vibrational Raman gain suppression. All pulses in (b) and (c) are normalized to a peak power equal to one.

Despite the apparent similarity of the steady-state and transient regimes, they exhibit some important differences. First, the connection of $Im\left[R\right]$ between the steady-state value ${\left|A^{P}\right|}^{2}T_{2}$ and the transient value $\int_{-\infty }^{t}{\left|A^{P}\left(\tau \right)\right|}^{2}\;d\tau ∼{\left|A^{P}\right|}^{2}△t_{p}$ shows that the transient Raman gain is smaller than the steady-state gain by a factor of △*t*_*p*_/*T*_2_. This leads to the potential existence of a Raman gain peak for an ultrashort pulse due to the reduced ImRtr or increased $\text{Re}\left[\kappa_{e}{\left|A^{P}\right|}^{2}+R_{\text{tr}}\right]$. In addition, monotonically increasing ImRtr with time results in not only stronger gain at the pulse trailing edge but also a varying gain shape throughout the pulse [Fig. 3(a)]. There is also a significant difference regarding where the Raman growth is the strongest. The Raman gain in the steady-state regime depends on pulse instantaneous intensity, whereas it relies on integrated pulse energy in the transient regime. This leads to the strongest Raman generation at the pulse center in the steady-state regime [Fig. 3(b)] but at the trailing edge in the transient regime [Fig. 3(c)].

It has long been stated that the transient Raman gain is proportional to the square root of the integrated pulse energy,^105,107,109,110^ which seems to contradict the results above. By ignoring pump depletion and keeping only the term for Raman growth, the Stokes governing equation [Eq. (7b)] can be solved analytically,^104,105,107^

$$\begin{array}{ll} A^{S}\left(z,t\right) & =A^{S}\left(0,t\right)+\kappa^{S}\omega^{S}R^{\text{coeff}}zA^{P}\left(t\right)\\ & \;\times \int_{-\infty }^{t}e^{-\gamma_{2}\left(t-\tau \right)}{\left(A^{P}\left(\tau \right)\right)}^{*}A^{S}\left(0,\tau \right)\frac{I_{1}\left(\sqrt{u}\right)}{\sqrt{u}}\;d\tau , \end{array}$$ (15)

where *I*_1_(*x*) is the modified Bessel function of the first kind; $u\left(t,\tau \right)=2\kappa^{S}\omega^{S}R^{\text{coeff}}z\left(U\left(t\right)-U\left(\tau \right)\right)$, where $U\left(t\right)=\int_{-\infty }^{t}{\left|A^{P}\left(\tau \right)\right|}^{2}\;d\tau$. By reasonably assuming *A*^*S*^ ∝ *A*^*P*^ and *γ*_2_ = 0, the Stokes field in Eq. (15) can be reduced to

$$\begin{array}{ll} A^{S}\left(z,t\right) & =A^{S}\left(0,t\right)I_{0}\left(\sqrt{u_{-\infty }}\right)\\ & \approx \left\{\begin{array}{cc} A^{S}\left(0,t\right)\left(1+\frac{1}{2}\kappa^{S}\omega^{S}R^{\text{coeff}}U\left(t\right)z\right),\; & u_{-\infty }\ll 1,\\ A^{S}\left(0,t\right)\frac{\text{exp}\left(\sqrt{u_{-\infty }}\right)}{\sqrt{2\pi \sqrt{u_{-\infty }}}},\; & u_{-\infty }\gg 1, \end{array}\right. \end{array}$$ (16)

where *u*_−*∞*_ = *u*(*t*, −*∞*) and the relation $\frac{\text{d}I_{0}\left(x\right)}{\text{d}x}=I_{1}\left(x\right)$ is employed. In studies of Raman gain, especially when the goal is to determine which type of SRS dominates, only the initial growth of each wave matters. Therefore, the Stokes growth is determined by the Raman gain 12κSωSRcoeffU(t)=κSωSImRtr (1 + *x* ≈ e^*x*^ as |*x*| ≪ 1), rather than $∼\;\sqrt{u_{-\infty }}$ in the opposite limit. This proportionality to the integrated pulse energy aligns with our finding that the transient Raman gain without parametric suppression from the anti-Stokes wave (or equivalently with |△*β*| ≫ 1) is directly proportional to the integrated pulse energy. Moreover, Eq. (15) shows that, in the limit of high and saturated gain, the Stokes wave is not affected by the exact form of its initial shape *A*^*S*^(0, *t*) because the rapid variations are averaged out by the integration, which results in coherent Stokes pulses that are free from spontaneous emission noise.^105^ In the low-gain or unsaturated regime or in the initial growth of Raman pulses, this statement is not valid; there, the Raman pulses are strongly influenced by spontaneous quantum noise and exhibit significant fluctuations.^166,167^

In transient Raman scattering with high and saturated gain, the Raman pulses tend to inherit the temporal phase of the pump pulse. This results in periodic temporal modulations of participating pulses,^105,106,109^ self-similarity in pulse evolutions,^182–184^ Kerr-induced Raman suppression, and Raman pulse compression. In this paragraph, we will specifically focus on the last two effects. Konyashchenko *et al.* have discussed the suppression of Raman generation,^128^ which arises from the nonlinear wave-vector mismatch of pump and Raman pulses that do not have flat-top intensity profiles. This Kerr-induced suppression of Raman generation from SPM and XPM occurs because different rates of spectral broadening detune the frequency difference between the pump and the Stokes pulses from the Raman transition frequency (△*ω* = *ω*^*P*^ − *ω*^*S*^ ≠ *ω*_*R*_), where Raman generation is most efficient. Mathematically, a differential nonlinear phase between the pump and Stokes pulses results in a reduction of the Raman temporal integral $R_{2;i,j,k}$, which contains either $A^{P}{\left(A^{i}\right)}^{*}$ (*i* = *S* or *AS*) or its complex conjugate. Since the pump pulse acquires a phase of $\omega^{P}\kappa^{P}\left(\kappa_{e}^{P}+\sum_{u}\frac{R_{u}^{\text{coeff}}}{\omega_{R_{u}}}\right){\left|A^{P}\right|}^{2}$ through SPM and SRS (contributed by $R_{1}$) and the Stokes/anti-Stokes pulse acquires $\omega^{i}\kappa^{i}\left(2\kappa_{e}^{i}+\sum_{u}\frac{5R_{u}^{\text{coeff}}}{4\omega_{R_{u}}}\right){\left|A^{P}\right|}^{2}$ through XPM and SRS (contributed by $R_{1}$ and $\text{Re}\left[R_{\text{tr}}\right]$), the difference is $\kappa \left[\left(\omega^{P}-2\omega^{i}\right)\kappa_{e}+\left(\omega^{P}-\frac{5}{4}\omega^{i}\right)\sum_{u}\frac{R_{u}^{\text{coeff}}}{\omega_{R_{u}}}\right]{\left|A^{P}\right|}^{2}$, assuming that *κ* and *κ*_*e*_ are frequency-independent. This differential nonlinear phase increment is eliminated when the expression within the square brackets is equal to zero, a condition that can only be met in Stokes generation. To avoid suppression of Raman generation by SPM or XPM for non-flat-top pulses, there must be no differential nonlinear phase accumulation between the pump and Stokes pulses. For vibrational SRS in hydrogen with ∼125-THz transition frequency, this condition can be met with a linearly polarized pump wavelength around 970–1000 nm, which almost aligns with that of established and readily available Yb-based laser systems. Neglect of the Raman-induced nonlinear phases that arise from $R_{1}$ and $\text{Re}\left[R_{\text{tr}}\right]$ leads to the conclusion that Raman generation is most effective at 1205 nm.^128^ As will be discussed below, SRS in gases induces significant nonlinear phases, even larger than the electronic-induced nonlinear phases (Fig. 7) and is thus non-negligible. Here, we treat Raman suppression using a simplified model for the differential nonlinear phase. The most-accurate treatment of Raman gain suppression will come from direct incorporation of nonlinear-phase effects in the Raman gain equation [Eq. (14)]; that will require generalization of the theory beyond the assumption of “flat-top” pulses, which remains for future work.

**FIG. 4.**
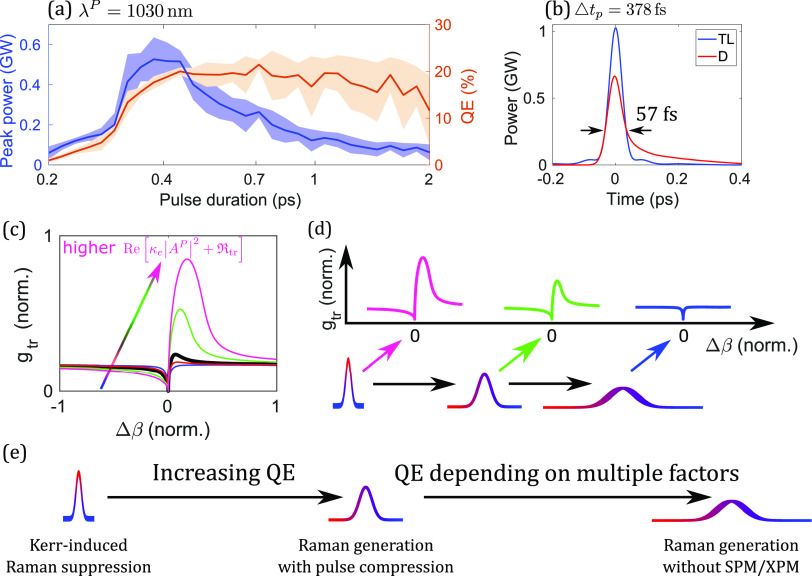
Nonlinear-phase effects on transient vibrational Stokes generation by chirped pulses with varying duration. Pulses at 1030 nm and with 500-*μ*J energy and 200-fs transform-limited pulse duration are launched into a 50-cm-long capillary, filled with H_2_ to 20-bar pressure. (a) Generation quantum efficiency (QE) and peak power of the dechirped output Stokes pulses, produced from the Raman process. Mean values (center lines) and 1*σ* standard deviations (shaded areas) are calculated from ten simulations for each duration. The *x* axis is plotted on logarithmic scale to expose the variation at small durations. (b) Temporal profile of the dechirped Stokes pulse with the highest peak power in (a). (TL: transform-limited pulse; D: dechirped pulse). (c) Transient Raman gain *g*_tr_ of a flat-top pulse, which ignores Kerr-induced Raman suppression from intensity-dependent SPM and XPM, with respect to wave-vector mismatch △*β* [Eq. (14)]. The arrow indicates the trend of increasing $\text{Re}\left[\kappa_{e}{\left|A^{P}\right|}^{2}+R_{\text{tr}}\right]$ (blue → red → black → green → pink) with fixed $Im\left[R_{\text{tr}}\right]$. (d) Varying Raman gain at the trailing edge of a pulse with varying chirped duration and a fixed pulse energy. (e) Summary of the influence of nonlinear phase modulations on the Raman process with different pulse durations in the transient regime.

The coherent nature of transient SRS can be illustrated by the simultaneous compression and Raman shifting of femtosecond-duration pulses. In numerical simulations, positively chirped pulses with a 200-fs transform-limited duration were launched into a H_2_-filled capillary. The pulse energy is fixed at 500 *μ*J for constant Raman gain (at huge |△*β*|); the varying chirped-pulse duration affects only the Kerr-induced nonlinear phase. Initially, the Stokes generation efficiency increases monotonically with increasing chirped duration due to weakening Kerr-induced Raman suppression [orange line in Fig. 4(a)]. When the gain is high and saturated, Raman pulses attempt to acquire the same phase as the pump to maximize the Raman integrals.^105^ This includes the acquisition of the nonlinear phase of the pump before the Kerr-induced suppression becomes significant. Spectral broadening through nonlinear phase accumulations can be used to compress pulses in a dispersive delay line. In this case, the SPM-induced nonlinear phase accumulated by the pump is transferred to the Stokes pulses, which can be compressed to durations about 3–15 times shorter than the input pump pulse [Fig. 4(b)]. In the transient Raman regime, Raman spectral narrowing occurs in the single-pulse approach, where only the trailing edge of the chirped pump pulse is transformed into Raman pulses and results in their narrower bandwidths than the pump’s. By chirping the pump appropriately, Raman spectral narrowing can be overcome by this pulse-compression effect. It has also been experimentally demonstrated by Konyashchenko *et al.*^129,130^ Figure 2 plots Raman-gain curves with a fixed real part and varying imaginary part of $\left(\kappa_{e}+R_{\text{ss}}\right){\left|A^{P}\right|}^{2}$ in the gain equation, which, in the transient regime, becomes $\left(\kappa_{e}{\left|A^{P}\right|}^{2}+R_{\text{tr}}\right)$. This is useful in visualizing the temporally varying Raman gain over a pulse [Fig. 3(a)]. On the other hand, it can be visualized differently with the varying real part and fixed imaginary part, which is the case of varying the chirp of a pulse here [Fig. 4(c)]. Because the real part of $\left(\kappa_{e}{\left|A^{P}\right|}^{2}+R_{\text{tr}}\right)$ depends on peak power, whereas its imaginary part on integrated pulse energy [Eqs. (13) and (14)], reducing the chirped-pulse duration but fixing the pulse energy creates the FWM peak in the Raman gain [Fig. 4(d)]. Therefore, with a proper wave-vector mismatch, Raman gain becomes stronger with reducing duration and the resulting higher peak power. This peak-power-dependent transient Raman-gain phenomenon is observed in the highly chirped regime in Fig. 4(a), where the efficiency starts to drop with increasing chirped-pulse duration. Fluctuations in generation efficiency arise from input-pulse shot noise and spontaneous Raman scattering, with increased significance observed in regimes of weaker Raman generation. However, in our example, heightened fluctuations are only evident in the long-pulse regime. In the short-pulse regime, Stokes generation is efficient due to the prominent Raman gain peak (with proper wave-vector mismatch) before Kerr-induced spectral detuning of pump and Stokes waves becomes significant. This initially efficient Raman generation quickly amplifies the pulse beyond the linear Raman regime where spontaneous emission noise dominates. After the initial generation, the Stokes pulse stops growing due to increased spectral detuning. On the other hand, in the long-pulse regime, slow Stokes generation, despite achieving a rather high efficiency after a long propagation, renders a Stokes pulse susceptible to spontaneous emission noise. Figure 4(e) summarizes the effects on Raman generation of varying nonlinear phase modulations: in the short-pulse regime, Kerr-induced Raman suppression dominates; increasing the pulse duration reduces the Kerr-induced suppression effect, enabling Raman pulse compression, and potentially introduces a rising Raman gain (if wave-vector mismatch meets where the gain peak is); further increasing the duration sees constant or dropping Raman gain based on the amount of wave-vector mismatch. More details about peak-power dependence, pulse compression, and quantum fluctuations are provided in Sec. 9 of the supplementary material.

In the steady-state regime, these coherent phenomena play out differently. Both Raman-enhanced SPM and nonlinear-phase-induced Raman suppression are observable in both the steady-state and the transient regimes [Eqs. (9a) and (13a)]. However, in the steady-state regime, spectral broadening can only result from the Raman process attempting to maintain a constant frequency difference *ω*^*P*^ − *ω*^*S*^ = *ω*_*R*_, where the highest Raman gain is, rather than from the transfer of the pump’s nonlinear phase as in the transient regime. If a Raman pulse originates from noise and lacks coherence, such incoherent spectral broadening does not lead to pulse compression. Since the steady-state Raman gain depends on $\left(\kappa_{e}+R_{\text{ss}}\right)$, determined only by materials, steady-state Raman gain in Raman-active gases has no FWM-induced gain peak and shows no varying gain shape with different chirped-pulse durations as in the transient regime.

### C. Impulsive regime (△*t*_*p*_ ≪ *T*_2_, △*t*_*p*_ ≪ *T*_*R*_)

**FIG. 5.**
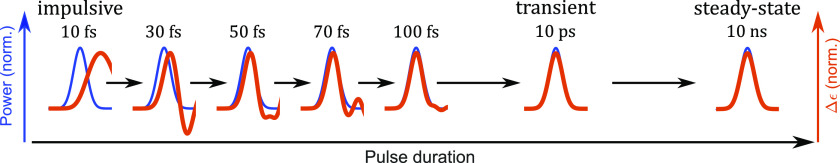
Normalized index change (orange) induced by rotational SRS (represented by △*ɛ*) of hydrogen in response to a pulse of varying pulse duration (blue). The oscillation period of the S(1) rotational transition is 56.8 fs. The index change is computed from $△\varepsilon \left(\vec{r},t\right)=\int_{-\infty }^{t}R\left(t-\tau \right){\left|\vec{E}\left(\vec{r},\tau \right)\right|}^{2}\;d\tau$, with $\vec{E}\left(\vec{r},t\right)$ being the analytic signal of the electric field.^185^

**FIG. 6.**
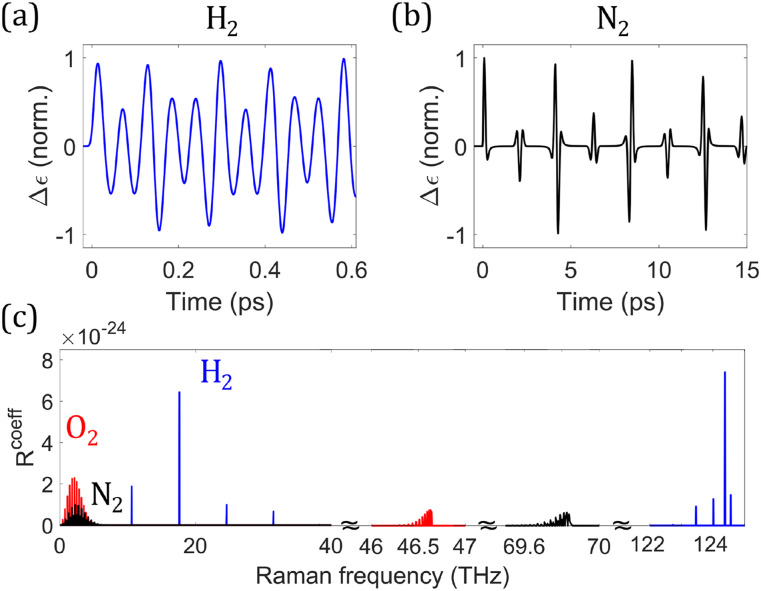
Impulsively excited index change in 1 bar of (a) H_2_ or (b) N_2_ due to rotational SRS. (c) Values of *R*^coeff^ for 1 bar of (blue) H_2_, (black) N_2_, and (red) O_2_ under the linearly polarized scalar assumption. The “revivals” in N_2_ and O_2_ at large time delays arise from beating of phonon waves from different rotational transitions,^186^ whereas in H_2_, the S(1) transition at 17.6 THz dominates, which leads to a constantly oscillating sinusoidal-like index wave.

In both the steady-state and transient regimes, the nuclear motions of a medium exhibit a response analogous to the electronic nonlinearity due to either a short phonon decay time or fast molecular oscillations. As a result, they both exhibit Kerr-like characteristics [Eqs. (9a) and (13a)] in addition to Raman gains [Eqs. (9b) and (13b)]. However, the response of the medium is no longer stationary if the pulse is shorter than the molecular oscillation period *T*_*R*_. The medium response is delayed relative to the pulse (Fig. 5). In addition to the delayed response, the medium is impulsively excited, which creates long-lived phonon waves after the pulse and allows for nonlocal interactions between pulses (Fig. 6).

To understand the SRS mechanism in the impulsive regime, we need to solve the Raman integrals [Eq. (8)], as in the other two regimes. The mathematical approximations in the impulsive regime are

$$\begin{array}{ll} & \int_{-\infty }^{t}\left[\sin\left(\omega_{R}\left(t-\tau \right)\right)\right]f\left(\tau \right)d\tau \\ & \;\approx \left\{\begin{array}{cc} \omega_{R}\left[△t\int_{-\infty }^{△t}\tilde{f}\left(x\right)dx-\int_{-\infty }^{△t}x\tilde{f}\left(x\right)dx\right],\; & 0<△t\leq △t_{p},\\ -\frac{\left|F\left[f\right]\left(-\omega_{R}\right)\right|}{C_{F}}\sin\left(-\omega_{R}t+\phi \right),\; & △t>△t_{p},\\ 0,\; & △t\leq 0, \end{array}\right. \end{array}$$ (17)

in which △*t* = *t* − *t*_*ℓ*_ and *t*_*ℓ*_ is where the leading edge of the pulse is. The substitution of variables *τ* = *t*_*ℓ*_ + *x* and $\tilde{f}\left(x\right)=f\left(t_{\ell }+x\right)$ is employed. *ϕ* is the phase of $F\left[f\right]\left(\omega_{R}\right)$. In contrast to the transient regime, the decomposition of the broadband total field into each component is not valid [Eq. (6)]. Instead, we focus on the evolution of the total field by substituting *A* for *A*^*P*^ in Eq. (7a). To gain insights into the evolution within the pulse, we make the flat-top assumption to simplify the equations and obtain, for 0 < △*t* ≤ △*t*_*p*_,

$$R_{1}\approx R^{\text{coeff}}{\left|A^{\text{peak}}\right|}^{2}\omega_{R}{\left(△t\right)}^{2}/2,$$ (18)

where ${\left|A^{\text{peak}}\right|}^{2}$ is the peak power of the flat-top pulse.

Due to the delayed response of the medium to the pulse, $R_{1}$ corresponds to the buildup of the Raman-induced index change, which leads to a time-dependent phase increment. This causes the pulse spectrum to red-shift $(△\omega =-\frac{\text{d}\left(△\phi \right)}{\text{d}\left(△t\right)}\propto -△t<0)\;$.^139^ Traditionally, red-shifting is treated as a consequence of impulsive phonon-wave generation after the pulse, whereas here it is explained by the nonlinear dynamics resulting from the index change. Greater red-shifting at the trailing edge of the pulse induces the negative chirp.^140^ If the dispersion is anomalous, the pulse will stretch temporally. On the other hand, in the normal-dispersion regime, this nonlinear phase will compete with the positive chirp from dispersion and SPM, potentially leading to pulse compression. However, in initial simulations, we find that SPM-induced chirp dominates over Raman-induced nonlinear phases. More investigations into this effect are thus required.

**FIG. 7.**
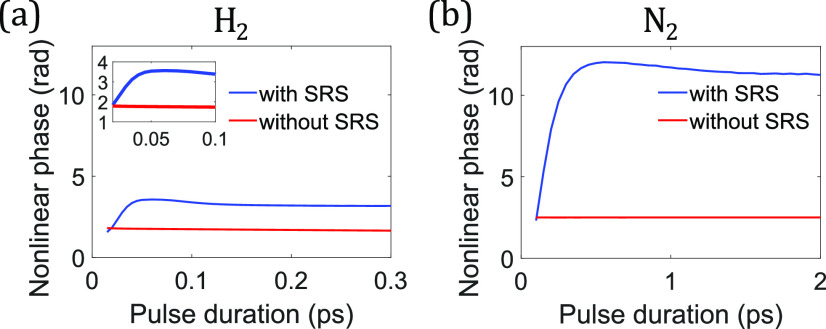
Accumulation of a nonlinear phase from numerical simulations with varying pulse duration in (a) H_2_ and (b) N_2_. A linearly polarized Gaussian pulse at 800 nm and with a fixed 6.67-MW peak power is launched into a 2-m-long anti-resonant fiber pressurized to 1 bar. The peak power is chosen so that the nonlinearity is sufficiently weak to avoid further amplitude-related Raman-gain effects, and the dispersion is artificially set to zero to isolate the nonlinear effects. The nonlinear phase *ϕ*_max_ is calculated from the spectral broadening of the pulse.^187,188^

The Raman-enhanced SPM effect in the transient regime, resulting from $R_{1}$ and $\text{Re}\left[R_{\text{tr}}\right]$, has been exploited to generate ultra-broadband continua and few-cycle pulses.^73,75^ However, with shorter pulses, the Raman-induced index changes undergo a transition from SPM and XPM in the transient regime [Eqs. (9a) and (13a)] to red-shifting in the impulsive regime [Eq. (18)], which diminishes the SPM enhancement. Figure 5 illustrates the transition from pulse-following (∝|*A*|^2^) to a rising index $\left[\propto {\left(△t\right)}^{2}\right]$ with reducing pulse duration. In H_2_, the transition occurs at around 50 fs, where the dominant S(1) rotational transition starts to become impulsive, while in N_2_, it occurs at 500 fs [Figs. 6(c) and 7]. Because N_2_ has more available Raman transitions, which are also at smaller frequencies [Fig. 6(c)], the Raman-induced SPM enhancement in N_2_ is about $\frac{11.3-2.50}{3.2-1.7}\approx 6$ times as strong as in H_2_, leading to the final $\frac{11.3}{3.2}\approx 3.5$ times stronger Raman-enhanced SPM. This effect has been studied with 30 and 280-fs pulses in H_2_^73^ and N_2_,^75^ respectively, and greater enhancements should be achievable by using pulses longer than the transition durations. On the other hand, simultaneous spectral broadening with red-shifting can produce few-cycle pulses at long wavelengths.^78^ The optimal pulse duration for this process lies between the transient and impulsive regimes, where the distinctive characteristics of Kerr and red-shifting effects both come into play. It is worth noting that the Raman-induced SPM is 4.5 times larger than the electronic-induced SPM in N_2_, while they have nearly the same magnitude in H_2_, values that are consistent with measurements of the total and electronic-induced nonlinear refractive indices.^186^

It is common to model the Raman response of a medium effectively as a single damped harmonic oscillator, with amplitude determined by the “Raman fraction” *f*_*r*_ of the total nonlinear response, which can be found from Fig. 7: $R\left(t\right)\propto \left(f_{\;r}n_{2}^{\text{total}}\right)e^{-\gamma_{2}t}\;\sin\left(\omega_{R}^{\text{eff}}t\right)$.^77–79,189,190^ We are now in position to examine the validity of the model while shedding some insights on Raman processes. This model is especially useful if the Raman response can be approximated as a harmonic oscillator with *strong damping*, which mitigates the potential numerical aliasing resulting from long-lived phonon waves. This is the case in N_2_ and O_2_. Due to their reviving indices, sufficiently short pulses essentially experience only the first spike of index change [Fig. 6(b)], which can be approximated by a highly damped harmonic oscillator. When the pulse becomes long enough to be affected by further reviving indices, this simplified model fails. In other words, the model operates by smearing the clustered sharp spectral Raman lines around 2 THz into one broadband Raman spectrum [Fig. 6(c)] and will fail when the smearing effect becomes invalid.

Weaker Raman-induced nonlinear phase accumulation can be advantageous for some processes. An example is SSFS, which has been widely studied in solid glass fibers.^191–197^ Since the soliton number is proportional to *γ*_eff_△*t*_*p*_,^198^ where *γ*_eff_ is the effective or total nonlinear coefficient, a pulse in the impulsive regime has a smaller soliton number than that in the transient regime due to the reduced pulse duration. A smaller soliton number mitigates energy loss during soliton fission and thus enhances the efficiency of generating the reddest Raman soliton during SSFS.^199^ Furthermore, the decrease in Raman enhancement of SPM leads to a reduction in *γ*_eff_, which also decreases the soliton number. The decrease can be as much as a factor of 4.5 times in N_2_, for example [Fig. 7(b)]. Finally, the reduced nonlinear phase is transformed to more-pronounced red-shifting through the rising Raman-induced index change [Eq. (18)]. Together, these factors make the SSFS a compelling option for wavelength-shifting in gases when it can be driven in the impulsive regime.

**FIG. 8.**
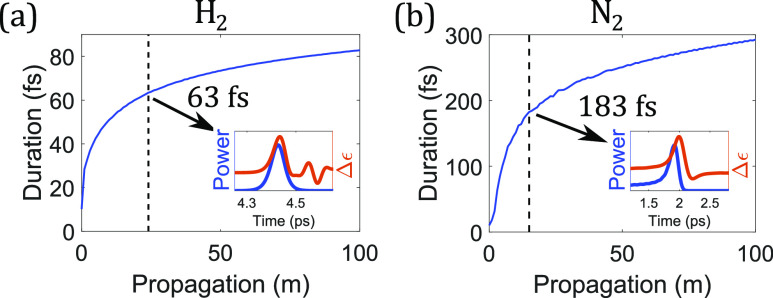
Evolutions of the (full-width-at-half-maximum) pulse duration during the SSFS process in a 100-m-long anti-resonant hollow-core fiber, filled with 10-bar (a) H_2_ or (b) N_2_. Black dashed lines are where the increase of the duration drops to 1% for every 1-m propagation. Insets are temporal profiles of the Raman-induced index changes (△*ɛ*; orange) and Raman solitons (blue) when the increase of the soliton duration significantly slows down (after black dashed lines). The injected linearly polarized fundamental solitons at 1030 nm have 10-fs duration, with 350-nJ energy in H_2_ and 270-nJ energy in N_2_ due to different dispersion and electronic nonlinearities.

The temporal behavior [Figs. 6(a) and 6(b)] of Raman-induced index change can play an important role in Raman processes. We continue with the example of the SSFS. In gases with ultra-narrowband Raman responses, only pulses in the impulsive regime have the required bandwidth for SSFS to occur. The Raman soliton continues to red-shift due to the rising Raman-induced index change. Moreover, the soliton duration increases and its bandwidth decreases due to the soliton area theorem.^198^ The SSFS significantly slows down when the Raman soliton becomes long enough that the Raman-induced index change starts to catch up with the pulse, and the nonlinear-phase-induced red-shifting ceases. As a result, the Raman-soliton duration is approximately bounded above by 1–2 times the duration of the first spike of the Raman-induced index change. In this situation, the Raman soliton can also become too narrowband for intrapulse SSFS to occur. In H_2_ where the dominant S(1) rotational transition has *T*_*R*_ = 57.8 fs [Fig. 6(a)], the soliton duration quickly evolves to 63 fs when the index starts to follow the pulse [Fig. 8(a)]. Similarly in N_2_, where the first spike is 118-fs-long [Fig. 6(b)], the soliton duration quickly evolves to 183 fs [Fig. 8(b)]. A slow increase of the duration follows due to a weakly delayed Raman-induced index change. In H_2_, the increase of the duration slows down also at its transition duration between two Raman regimes, where SSFS basically stops [Fig. 7(a)]. However, in N_2_, it slows down at a duration smaller than its transition duration of 500 fs [Fig. 7(b)]. Since the duration of temporal spikes of Raman response is inversely correlated with the collective spectral width of clustered Raman responses (as in N_2_ and O_2_), the ability of pulse-following is determined by not only the spectral positions of clustered Raman responses but also their collective spectral width. Higher frequencies and larger collective spectral width of clustered Raman responses correspond to a sharper temporal spike of the overall Raman response, leading to a shorter soliton duration after SSFS. On the contrary, since only one S(1) rotational transition dominates in H_2_, there is no such thing as collective spectral width and only the Raman frequency determines the temporal behavior of the Raman response that affects the Raman-soliton duration.

One of the most important features of impulsive SRS is its ability to create long-lasting phonon waves [Figs. 6(a) and 6(b)], which allows for nonlocal interactions between temporally separated pulses. This phenomenon has been extensively studied in situations with either two ultrashort pulses in the impulsive regime^91–95,101,131–143,200,201^ or two long pulses in the transient regime.^86–90^ However, a general description of this process covering pulses of all time scales has not been reported. Investigation of multi-pulse interactions through phonons can provide deeper understanding of the underlying physics and help optimize applications such as ultrafast time-resolved spectroscopy and controllable wavelength conversion. The following discussion builds on our initial attempts toward the unified theory of SRS.^90^ From Eq. (17), the Raman response to a short pulse is

$$R_{1}\approx -\frac{\left|F\left[|A_{1}{|}^{2}\right]\left(-\omega_{R}\right)\right|}{C_{F}}\sin\left(-\omega_{R}t+\phi \right),\;△t>△t_{p}.$$ (19)

**FIG. 9.**
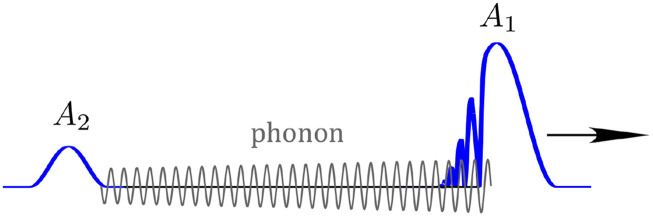
Double-pulse approach to Raman generation that avoids Raman spectral narrowing. The first pulse excites the phonon waves, which scatter the second pulse starting with its leading edge so that the entire pulse undergoes Raman generation, rather than only the trailing edge that results in Raman spectral narrowing for a chirped pulse.

The strength of the excited phonon waves is governed by the power spectral density $F\left[|A_{1}{|}^{2}\right]$ at −*ω*_*R*_. Even if the pulse is initially in the transient regime, the generated Stokes or anti-Stokes waves can become sufficiently strong to induce beating with the pump. Short pulses that result from the beating then impulsively excite phonon waves.^73^ This is the basis of a technique designed to avoid Raman spectral narrowing and generate femtosecond Stokes pulses with excitation pulses in the transient regime (Fig. 9).^86–90^

**FIG. 10.**
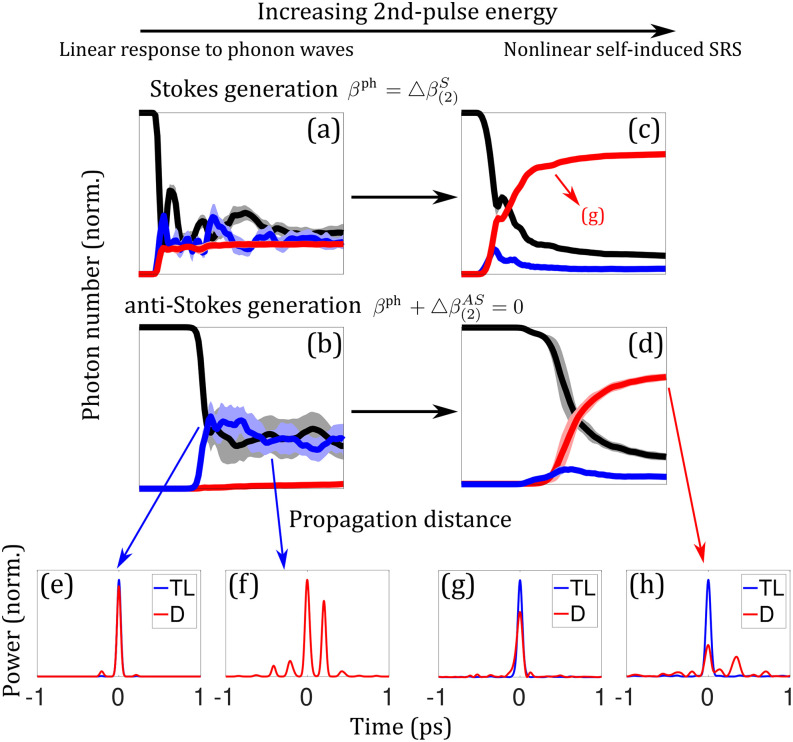
Effects of phonon dynamics in the transient regime. Two 50-fs pulses of different wavelengths are chirped to 10-ps duration and are launched into a 1-m-long capillary filled with 20-bar H_2_. Their wavelengths are chosen to yield the process of interest, with the varying first-pulse wavelength and fixed second-pulse 2-*μ*m wavelength. Because of the unique S-shaped dispersion curve of gas-filled HCF, pumps of different wavelengths create different phonon wave vector *β*^ph^.^64,90^ Evolutions of photon numbers of Stokes (red), pump (black), and anti-Stokes (blue) waves under different wave-vector-matching conditions are displayed for the cases of weak (10 *μ*J) and strong (5 mJ) second-pulse energy; the first pulse is fixed at 2 mJ. (a) and (c) satisfy the phonon amplification condition, where the first pulse is at 0.7 *μ*m, while (b) and (d) satisfy the phonon absorption condition, where the first pulse is at 1.09 *μ*m. The second pulse is weak in (a) and (b), while it is sufficiently strong to induce its own SRS in (c) and (d). Mean values (center lines) and 1*σ* standard deviations (shaded areas) are calculated from ten repeated simulations. (e) and (f) The temporal profiles of the dechirped anti-Stokes pulses from (b); in (f), the pulse quality is degraded by back-conversion. (f) The result from one of the ten simulations, which fluctuates significantly due to the fluctuating phonon-beating pattern. (g) and (h) The temporal profiles of the dechirped Stokes pulses from (c) and (d), respectively. The Stokes pulse in (g) has more than twice the peak power as that in (h). Despite being not shown here, the pulse quality of the dechirped Stokes pulse in (a) is the same as that in (g) from (c) (TL: transform-limited pulse, D: dechirped pulse).

Phonons can interact with light pulses through multiple mechanisms. If the second pulse is weak and long such that it is in the transient Raman regime [Figs. 10(a) and 10(b)], it can undergo either Stokes or anti-Stokes generation depending on the wave-vector mismatch,

$$\partial_{z}A_{2}^{S}\left(z,t\right)=-\frac{\omega^{S}\kappa^{S}C}{2}e^{-\gamma_{2}△t}A_{2}^{P}e^{-i\left[\left(\beta^{\text{ph}}-△\beta_{\left(2\right)}^{S}\right)z+\phi \right]},$$ (20a)

$$\partial_{z}A_{2}^{AS}\left(z,t\right)=\frac{\omega^{AS}\kappa^{AS}C}{2}e^{-\gamma_{2}△t}A_{2}^{P}e^{i\left[\left(\beta^{\text{ph}}+△\beta_{\left(2\right)}^{AS}\right)z+\phi \right]},$$ (20b)

where $△\beta_{\left(j\right)}^{i}=\beta_{j}^{P}-\beta_{j}^{i}$ is the difference of the propagation constants of the *i* (Stokes or anti-Stokes) waves in the *j*th pulse, *β*^ph^ is the wave vector of the excited phonon waves, and *C* is the strength of the phonon waves. [See Sec. 10 of the supplementary material for the derivation of Eq. (20).] This wave-vector-matching effect can be treated as phonon amplification for Stokes generation and phonon absorption for anti-Stokes generation.^64,90^ Phonon amplification is the strongest when the incoming phonons perfectly seed the process by having the same wave vector as the generated phonons, $\beta^{\text{ph}}=\left(\beta_{\left(2\right)}^{\text{ph}}\equiv △\beta_{\left(2\right)}^{S}\right)$. On the other hand, phonon absorption is most efficient if the incoming phonons satisfy $\beta^{\text{ph}}+△\beta_{\left(2\right)}^{AS}=0$. The response of the second pulse to the incoming phonons is linear owing to the absence of $A_{2}^{S/AS}$ on the right-hand side of Eq. (20) and negligible pump depletion. If both wave-vector-matching relations are simultaneously satisfied, Stokes and anti-Stokes waves experience growth; this occurs for $△\beta_{\left(2\right)}^{S}+△\beta_{\left(2\right)}^{AS}=△\beta_{\left(2\right)}=0$. This observation highlights the fact that phonon waves play a linear driving role in the SRS process and thus can overcome (nonlinear) Raman gain suppression at △*β*_(2)_ = 0. During the phonon-absorption process [$\beta^{\text{ph}}+△\beta_{\left(2\right)}^{AS}=0$, but, in general, $\beta^{\text{ph}}\neq △\beta_{\left(2\right)}^{S}$], phonons can still, to some extent, drive the generated anti-Stokes waves back to the pump through Stokes scattering. This results in oscillatory energy exchange between the anti-Stokes and pump waves [Fig. 10(b)]. Furthermore, the beating of phonon waves resulting from multiple Raman processes in gases creates non-uniform back-conversion of the anti-Stokes pulse to the pump, which degrades the quality of the anti-Stokes pulse [Figs. 10(e) and 10(f)]. Since the second pulse is weak, one might expect the generated Raman pulses to exhibit up to ∼100% energy fluctuations.^166,167^ However, this is not observed in phonon amplification [Fig. 10(a)] and the initial evolution of phonon absorption [Fig. 10(b)]. Existing coherent phonons linearly drive the Raman generation process and effectively dominate noisy thermal phonons and vacuum fluctuations, which stabilizes Raman pulses. This process enables quantum-state-preserving frequency conversion,^66^ for example. On the other hand, significant fluctuations are observed in the latter evolution of phonon absorption. This results from oscillatory evolutions between the anti-Stokes and pump waves, caused by phonon-induced back-conversion. Since the temporal pattern of phonon beating is strongly influenced by quantum fluctuations, back-conversion induces significant fluctuations to the Raman processes. Things turn out differently if the second pulse is strong. In this situation, self-induced SRS dominates [Figs. 10(c) and 10(d)]. The phonon-driven Stokes wave acts as the seed for the latter SRS process [Figs. 10(c) and 10(g)], but the growth of the phonon-driven anti-Stokes wave is suppressed by the aforementioned back-conversion, and eventually, the Stokes wave dominates [Fig. 10(d)]. Despite the domination of the Stokes process under the condition of phonon absorption $\left(\beta^{\text{ph}}+△\beta_{\left(2\right)}^{AS}=0\right)$ in the strong-pump situation, the non-uniform anti-Stokes back-conversion process in the initial process modifies the phase structure of the pump wave, leaving the latter-generated Stokes pulse temporally structured [Fig. 10(h)]. Unlike the low-energy regime where significant fluctuations exist in the phonon-absorption process, large nonlinearity of the self-induced SRS stabilizes evolutions and all participating pulses regardless of which phonon process to occur.

**FIG. 11.**
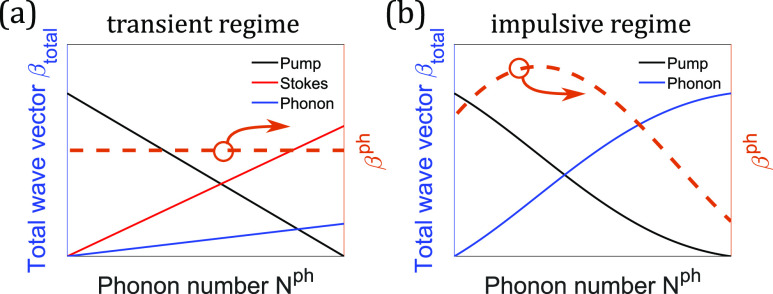
Evolutions of phonon wave vector *β*^ph^ during the SRS process in two different Raman regimes. Black, red, and blue lines represent total wave vectors of pump, Stokes, and phonons, respectively. Since the total momentum should be conserved, phonon wave vector is the difference between the pump and the Stokes waves in the transient regime: *β*_total_ = *N*^*P*^*β*^*P*^ + *N*^*S*^*β*^*S*^ + *N*^ph^*β*^ph^, where *N*^*P*^ + *N*^*S*^ + *N*^ph^ = *N*_total_ represents the number of photons/phonons. In the impulsive regime, no discrete Stokes wave is generated and the pump pulse shifts to the red with varying propagation constant: $\beta_{\text{total}}=N^{P}\beta^{P}+\sum_{i=1}^{N^{\text{ph}}}\beta^{\text{ph},i}$, where *N*^*P*^ + *N*^ph^ = *N*_total_. In (b), *β*^*P*^ ∼ cos(*N*_total_ − *N*^*P*^) is assumed to artificially create a varying *β*^*P*^ to simulate the red-shifting of the pump. Because of the red-shifting process, phonon wave vector is not a constant, in contrast to the transient regime.

Control of the Raman processes through the wave-vector matching relies on a fixed phonon wave vector *β*^ph^. This assumption holds only when phonons are excited through Stokes generation with a long pulse in the transient regime, resulting in phonons with a fixed wave vector equal to the difference between the pump and the Stokes fields, $\beta^{\text{ph}}=△\beta_{\left(1\right)}^{S}=\beta_{\left(1\right)}^{P}-\beta_{\left(1\right)}^{S}$ [Fig. 11(a)]. If the excitation pulse is short enough to enter the impulsive regime, the phonon wave vector is no longer fixed, but is instead determined by the nonlinear red-shifting process [Fig. 11(b)]. Since the phonon wave vector is not constant, the wave-vector-matching condition cannot be consistently met throughout the evolution, which results in an uncontrolled scenario of Stokes and anti-Stokes generation from the second pulse. This knowledge not only directs us toward controllable Raman generation through nonlocal phonon interactions but also offers more information of various Raman processes previously investigated, such as the phonon amplification conducted by Bustard *et al.*^202^ If phonons are impulsively excited by an ultrashort pulse, the upcoming pulse for amplifying the phonons with transient SRS must be sufficiently strong to guarantee the Stokes generation.

The story is different when the second pulse is in the impulsive Raman regime. In this situation, there is no discrete generation of Stokes or anti-Stokes fields by the second pulse. However, control of the delay between the two pulses permits for the control of the index change experienced by the second pulse. This can lead to controlled red-shifting through the rising index, as observed with $R_{1}$ in the discussed impulsive regime [Eq. (18)], or blue-shifting for the falling index.^92–95^

## IV. VECTOR PROPERTIES OF RAMAN RESPONSES

**FIG. 12.**
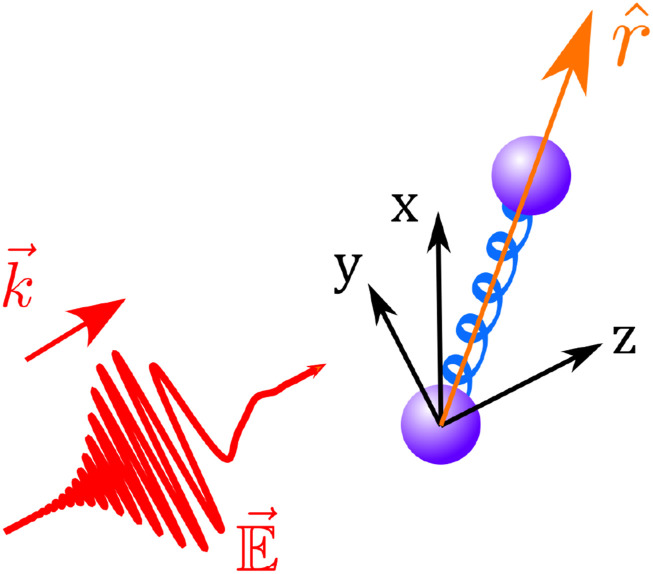
Illustration of spherical coordinates, where the diatomic molecular axis is aligned along $\hat{r}$ and the field propagates $\left(\vec{k}\right)$ along $\hat{z}$. The field is polarized in the *xy*-plane.

Vector (i.e., polarization) effects in SRS from molecules have been largely neglected. Vibrational SRS is almost isotropic and thus exhibits good consistency between experiments and scalar models.^203,204^ The isotropy results from $\left\langle \vec{E}\cdot \hat{r}\hat{r}\cdot \vec{E}\right\rangle \approx |\vec{E}{|}^{2}/3$ in the perturbative regime, where $\vec{E}$ and $\hat{r}$ are shown in Fig. 12. (Details are in Sec. 5 of the supplementary material.) Rotational SRS in gases requires a vector model due to its anisotropic nature arising from the exchange of angular momentum. Although there are a few prior works deriving the scalar model for rotational SRS in gases, they neglect its anisotropic nature.^76,186,190,205–208^ Here, we aim to address this gap by introducing a vector UPPE for gases.

There is a long history of investigation of rotational SRS in gases. Since Yoshikawa and Imasaka proposed the idea of phase-locking an SRS-induced multi-frequency spectrum to produce femtosecond pulses,^209^ simultaneous generation of multiple orders of Stokes/anti-Stokes waves has received substantial attention.^72,76,141,151–153,210–213^ One of the promising approaches is through molecular phase modulation, which requires impulsive excitation of gas molecules through SRS.^63,92,141,151–153,210,212–215^ While vibrational SRS can potentially produce sub-femtosecond pulses, rotational SRS is usually preferred in experiments because the longer period makes it easier to reach the impulsive regime. In addition, it can fill the spectral gap between vibrational Stokes/anti-Stokes wavelengths to create a smooth supercontinuum^76^ for potential generation of a single ultrashort pulse. It has long been known that rotational SRS is sensitive to the polarization of the field. A circularly polarized field preferentially drives rotational transitions, while linearly polarized light preferentially drives vibrational transitions.^84,203,211^ How these two scattering processes compete under different polarization conditions, and how to quantify the outcomes, have remained unclear. Rotational SRS, particularly in N_2_, is commonly observed with a linearly polarized field,^37–40,46,47,68,72,75–78,90,95,138,143,151,153,201,211,214^ but how its anisotropic nature affects the polarization and the subsequent nonlinear dynamics is unknown.

### A. Derivation of the vector UPPE

Derivation of the vector UPPE begins with the (real-valued) polarization $\left\langle {\vec{P}}_{R,N_{g}}^{\text{rot}}\right\rangle$, averaged over microscopic molecular orientations and summed over many molecules (*N*_*g*_ is the number density of gas molecules in 1/m^3^). Here, we consider only diatomic molecules to simplify the derivation (Fig. 12), but an analytic expression should also be possible for more-complicated molecules by following the same process.

Here, we outline the derivation of the rotational Raman response; details are included in Sec. 6 of the supplementary material. The derivation generalizes the perturbative density-matrix approach of Chen *et al.*^207^ by including the tensor polarizability $\alpha =\alpha_{\|}\hat{r}\hat{r}+\alpha_{⊥}\left(\hat{\theta }\hat{\theta }+\hat{\phi }\hat{\phi }\right)$ of a diatomic molecule.^127^ With the identity relation $\hat{r}\hat{r}+\hat{\theta }\hat{\theta }+\hat{\phi }\hat{\phi }=I$, the Raman-induced polarization ${\vec{P}}_{R}^{\text{rot}}=\alpha \cdot \vec{E}$ from a single molecule becomes

$${\vec{P}}_{R}^{\text{rot}}=△\alpha \hat{r}\hat{r}\cdot \vec{E}+\alpha_{⊥}\vec{E},$$ (21)

where △*α* = *α*_‖_ − *α*_⊥_ is the polarizability anisotropy and $\vec{E}$ is the real-valued electric field. The SRS-induced change of populations in different energy levels can be obtained as a perturbation of the density matrix,^216^

$$\rho_{ab}^{\left(1\right)}=-\frac{i}{\hbar }\int_{-\infty }^{t}{\left[H_{\text{int}}\left(\tau \right),\rho^{\left(0\right)}\right]}_{ab}e^{\left(\gamma_{ab}+i\omega_{ab}\right)\left(\tau -t\right)}\;\mathrm{d\tau},\;$$ (22)

where the density matrix ***ρ*** = ***ρ***^(0)^ + ***ρ***^(1)^ with the unperturbed density matrix ***ρ***^(0)^ satisfying the Boltzmann distribution.^217^ **H**_int_ is the perturbed Hamiltonian of the system in the interaction picture. *γ*_*ab*_ and *ω*_*ab*_ = *ω*_*a*_ − *ω*_*b*_ are the dephasing rate and frequency of the *a* ↔ *b* transitions, respectively. The solution to the density matrix allows for the determination of the microscopic-orientation-averaged SRS-induced polarization through

$$\begin{array}{ll} \left\langle {\vec{P}}_{R}^{\text{rot}}\right\rangle & =△\alpha \left\langle \hat{r}\hat{r}\cdot \vec{E}\right\rangle +\alpha_{⊥}\vec{E}\\ & =△\alpha \mathrm{Tr}\left(\rho \hat{r}\hat{r}\cdot \vec{E}\right)+\alpha_{⊥}\vec{E}, \end{array}$$ (23)

where the microscopic orientational averaging is applied over multiple rotational states.

The unperturbed part of Eq. (23) can be solved with a simple integral over solid angle. By observing that ${\left\langle \hat{r}\hat{r}\cdot \vec{E}\right\rangle }^{\left(0\right)}={\left\langle \frac{\sin^{2}\;\theta }{2}\right\rangle }^{\left(0\right)}\vec{E}=\frac{\vec{E}}{2}\left(\frac{1}{4\pi }\int \sin^{2}\;\theta \;d\Omega \right)=\frac{\vec{E}}{3}$, the unperturbed polarization ${\left\langle {\vec{P}}_{R}^{\text{rot}}\right\rangle }^{\left(0\right)}=\frac{\alpha_{\|}+2\alpha_{⊥}}{3}\vec{E}$ corresponds to the linear susceptibility, which is usually replaced by a Sellmeier equation.^218–221^

To solve the perturbed part of Eq. (23), we need $\rho_{ab}^{\left(1\right)}$, which leads us to first finding $H_{\text{int}}\left(\vec{r},t\right)$,

$$\begin{array}{ll} H_{\text{int}} & =-\int \vec{E}\cdot \;d{\vec{P}}_{R}^{\text{rot}}\\ & =-\frac{△\alpha }{2}\vec{E}\cdot \hat{r}\hat{r}\cdot \vec{E}-\frac{\alpha_{⊥}}{2}|\vec{E}{|}^{2}. \end{array}$$ (24)

Therefore,

$${\left[H_{\text{int}}\left(\tau \right),\rho^{\left(0\right)}\right]}_{ab}=-\left(\rho_{b}^{\left(0\right)}-\rho_{a}^{\left(0\right)}\right)\frac{△\alpha }{2}{\left(\vec{E}\cdot \hat{r}\hat{r}\cdot \vec{E}\right)}_{ab},$$ (25)

where $\rho_{ab}^{\left(0\right)}=\rho_{a}^{\left(0\right)}\delta_{ab}$ and *δ*_*ab*_ is the Kronecker delta function. Equation (22) then becomes

$$\rho_{ab}^{\left(1\right)}=\frac{i}{\hbar }\left(\rho_{b}^{\left(0\right)}-\rho_{a}^{\left(0\right)}\right)\frac{△\alpha }{2}\int_{-\infty }^{t}{\left[\vec{E}\left(\tau \right)\cdot \hat{r}\hat{r}\cdot \vec{E}\left(\tau \right)\right]}_{ab}e^{\left(\gamma_{ab}+i\omega_{ab}\right)\left(\tau -t\right)}\;d\tau ,$$ (26)

where $\vec{E}\cdot \hat{r}\hat{r}\cdot \vec{E}$ plays the most important role as it determines the interaction between the gas molecule and the field. While $\left\langle \vec{E}\cdot \hat{r}\hat{r}\cdot \vec{E}\right\rangle =|\vec{E}{|}^{2}/3+{△}_{\hat{r}\hat{r}}$, where ${△}_{\hat{r}\hat{r}}\ll |\vec{E}{|}^{2}/3$, the deviation from the equilibrium value $|\vec{E}{|}^{2}/3$ results in rotational SRS. The small perturbation explains why vibrational SRS weakly depends on the molecular orientation and is thus isotropic. Prior works that assume linearly polarized fields obtain a simple $|\vec{E}{|}^{2}\cos^{2}\left(\frac{\pi }{2}-\theta \right)$ dependence for $\vec{E}\cdot \hat{r}\hat{r}\cdot \vec{E}$,^68,207^ where *θ* is the angle between the molecule $\hat{r}$ and the *z* axis, and the field is polarized in the *xy*-plane. The $\cos^{2}\left(\frac{\pi }{2}-\theta \right)$ dependence underlies what is referred to as “molecular alignment” in response to an external field.^75,77,138,143,190,201,207^ However, the response becomes complicated when $\vec{E}$ is not linearly polarized. Similarly, we need $\hat{r}\hat{r}\cdot \vec{E}$ to solve Eq. (23). After a lengthy derivation, we obtain

$$\vec{E}\cdot \hat{r}\hat{r}\cdot \vec{E}=\frac{1}{4}\left[2C_{0}|B{|}^{2}+C_{-2}{\left(B^{*}\right)}^{2}+C_{2}B^{2}\right],$$ (27a)

$$\hat{r}\hat{r}\cdot \vec{E}=\frac{1}{2}\left[\left(C_{0}B+C_{-2}B^{*}\right){\hat{\varepsilon }}_{+}\left(C_{0}B^{*}+C_{2}B\right){\hat{\varepsilon }}_{-}\right],$$ (27b)

in which $B=E_{+}+E_{-}^{*}$. The analytic signal of the real-valued electric field $\left[\vec{E}=\frac{1}{2}\left(\vec{E}+\text{c.c.}\right)\right]$ is $\vec{E}=E_{+}{\hat{\varepsilon }}_{+}+E_{-}{\hat{\varepsilon }}_{-}$, where ${\hat{\varepsilon }}_{\pm }=\frac{\hat{x}\pm i\hat{y}}{\sqrt{2}}$ is the basis of circular polarization. *C*_0_, *C*_−2_, and *C*_2_ are constants with spherical harmonics, $Y_{2}^{0}\left(\theta \right)$, $Y_{2}^{-2}\left(\theta ,\phi \right)$, and $Y_{2}^{2}\left(\theta ,\phi \right)$, respectively. Note that $|\vec{E}{|}^{2}=\frac{1}{2}|B{|}^{2}\approx \frac{1}{2}|E{|}^{2}$.

With ***ρ***^(1)^ from Eq. (26), we can solve for the orientational average of the perturbed part of Eq. (23), ${\left\langle \hat{r}\hat{r}\cdot \vec{E}\right\rangle }^{\left(1\right)}$,

$$\begin{array}{ll} {\left\langle \hat{r}\hat{r}\cdot \vec{E}\right\rangle }^{\left(1\right)} & =\mathrm{Tr}\left(\rho^{\left(1\right)}\hat{r}\hat{r}\cdot \vec{E}\right)=\underset{a,b}{\sum }\rho_{ab}^{\left(1\right)}{\left(\hat{r}\hat{r}\cdot \vec{E}\right)}_{ba}\\ & =\underset{JM,J^{\prime }M^{\prime }}{\sum }\rho_{JM,J^{\prime }M^{\prime }}^{\left(1\right)}\left\langle J^{\prime }M^{\prime }|\hat{r}\hat{r}\cdot \vec{E}|JM\right\rangle , \end{array}$$ (28)

which includes the product of $\left\langle JM|\vec{E}\cdot \hat{r}\hat{r}\cdot \vec{E}|J^{\prime }M^{\prime }\right\rangle$ and $\left\langle J^{\prime }M^{\prime }|\hat{r}\hat{r}\cdot \vec{E}|JM\right\rangle$ [Eq. (26)]. With the help of Gaunt coefficients^222,223^ and the Racah formula,^185,224^ we obtain the following relation for *C*_*μ*_ (*μ* = 0, ±2) [Eq. (27)], which is used to solve for the product:

$$\begin{array}{ll} & \underset{M,M^{\prime }}{\sum }\left\langle JM|C_{\mu }|J^{\prime }M^{\prime }\right\rangle \left\langle J^{\prime }M^{\prime }|C_{\nu }|JM\right\rangle \\ & \;=\frac{3\left(J+1\right)\left(J+2\right)}{10\left(2J+3\right)}\sigma \delta_{\mu ,-\nu }\left(\delta_{J+2,J^{\prime }}+\delta_{J-2,J^{\prime }}+C^{\prime }\delta_{J,J^{\prime }}\right), \end{array}$$ (29)

where *σ* = 1/9 if *μ* = *ν* = 0 or 2/3 if |*μ*| = |*ν*| = 2. *C*′ is a constant in a term that will not be considered further because only off-diagonal terms contribute to SRS. Using the fact that ${\left[\rho_{ab}^{\left(1\right)}{\left(\hat{r}\hat{r}\cdot \vec{E}\right)}_{ba}\right]}^{*}=\rho_{ba}^{\left(1\right)}{\left(\hat{r}\hat{r}\cdot \vec{E}\right)}_{ab}$ in the summation and with Eqs. (27)–(29), we obtain the analytic signal of ${\vec{P}}_{R,N_{g}}^{\text{rot}}=\frac{1}{2}\left({\vec{P}}_{R,N_{g}}^{\text{rot}}+\text{c.c.}\right)$,

$$\begin{array}{ll} {\vec{P}}_{R,N_{g}}^{\text{rot}} & =N_{g}\frac{{\left(△\alpha \right)}^{2}}{60\hbar }\underset{J}{\sum }\left(\rho_{J}^{\left(0\right)}-\rho_{J+2}^{\left(0\right)}\right)\frac{\left(J+1\right)\left(J+2\right)}{\left(2J+3\right)}\\ & \;\times \left\{\left\{\left(R_{J}^{\prime }*|B{|}^{2}\right)E_{+}+6\left[R_{J}^{\prime }*\left(E_{+}E_{-}^{*}\right)\right]E_{-}\right\}{\hat{\varepsilon }}_{+}\right.\\ & \;+\left.\left\{\left(R_{J}^{\prime }*|B{|}^{2}\right)E_{-}+6\left[R_{J}^{\prime }*\left(E_{+}^{*}E_{-}\right)\right]E_{+}\right\}{\hat{\varepsilon }}_{-}\right\}, \end{array}$$ (30)

where $R_{J}^{\prime }\left(t\right)=\Theta \left(t\right)e^{-\gamma_{2}t}\;\sin\left(\omega_{J+2,J}t\right)$. The dephasing rate *γ*_*J*+2,*J*_ = *γ*_2_ is assumed to be the same for all transitions.

Equation (30) is the vector rotational Raman response, but it is not in a form that provides straightforward information about its isotropy and anisotropy. In addition, its formulation with circularly polarized fields necessitates a vector transformation if the field is not circularly polarized, hindering us from straightforward understanding of the nonlinear dynamics of different polarizations. With the help of Eq. (1b), we can identify the isotropic and anisotropic parts of the rotational response [Eq. (31)]. Including the contributions of both the vibrational^68^ and rotational SRS, the final Raman response functions in the vector UPPE [Eq. (1)] become

$$\frac{1}{2}R_{a}=R_{a}=R^{\text{vib}}-2R^{\text{rot}},\;\;\frac{1}{2}R_{b}=R_{b}=6R^{\text{rot}},\;$$ (31a)

where

$$\begin{array}{rcl} R^{\text{vib}}\left(t\right) & \;= & \;\Theta \left(t\right)N_{g}\frac{{\left(\frac{\text{d}\alpha }{\text{d}Q}\right)}_{0}^{2}}{4\mu }e^{-\gamma_{2}^{\text{vib}}t}\sum_{J}\left(2J+1\right)\rho_{J}^{\left(0\right)}\frac{1}{\omega_{1,J;0,J}}\sin\left(\omega_{1,J;0,J}t\right),\\ R^{\text{rot}}(t) & \;= & \;\Theta \left(t\right)N_{g}\frac{{\left(△\alpha \right)}^{2}}{60\hbar }e^{-\gamma_{2}^{\text{rot}}t}\sum_{J}\left(\rho_{J}^{\left(0\right)}-\rho_{J+2}^{\left(0\right)}\right)\\ & & \times \frac{\left(J+1\right)\left(J+2\right)}{\left(2J+3\right)}\sin\left(\omega_{0,J+2;0,J}t\right). \end{array}$$ (31b)

${\left(\frac{\text{d}\alpha }{\text{d}Q}\right)}_{0}$ is the polarizability derivative at equilibrium, and *μ* is the reduced mass of the molecule (*μ* = *m*/2 for homonuclear diatomic molecules, where *m* is the atomic mass). $\gamma_{2}^{\text{vib}}$ and $\gamma_{2}^{\text{rot}}$ are the dephasing rates of vibrational and rotational transitions, respectively. $\omega_{\nu_{2},J_{2};\nu_{1},J_{1}}=\omega_{\nu_{2},J_{2}}-\omega_{\nu_{1},J_{1}}$ is the angular frequency of the transition between states (*ν*_1_, *J*_1_) and (*ν*_2_, *J*_2_). *ν* and *J* are the vibrational and rotational quantum numbers, respectively.

To facilitate the subsequent analysis, we explicitly show the nonlinear terms under circular [Eq. (32a)] and linear bases [Eq. (32b)]. For a linearly polarized field [*A*_*y*_ = 0 in Eq. (32b)], the rotational Raman response is 4*R*^rot^, which has a coefficient $\frac{4}{60}=\frac{1}{15}$. This is consistent with the derivation of the scalar linearly polarized UPPE,^68^

$$\begin{array}{ll} \hat{N}A_{+} & =i\omega \kappa \left\{\kappa_{e}F\left[\left(\frac{2}{3}|A_{+}{|}^{2}+\frac{4}{3}|A_{-}{|}^{2}\right)A_{+}\right]\right.\\ & \;+\left.F\left[\left[\left(R^{\text{vib}}+R^{\text{rot}}\right)*\left(|A_{+}{|}^{2}+|A_{-}{|}^{2}\right)\right]A_{+}\right.\right.\\ & \;+\left.\left.6\left[R^{\text{rot}}*\left(A_{+}A_{-}^{*}\right)\right]A_{-}\right]\right\}, \end{array}$$ (32a)

$$\begin{array}{ll} \hat{N}A_{x} & =i\omega \kappa \left\{\kappa_{e}F\left[\left(|A_{x}{|}^{2}+\frac{2}{3}|A_{y}{|}^{2}\right)A_{x}+\frac{1}{3}A_{y}^{2}A_{x}^{*}\right]\right.\\ & \;+\left.F\left[\left[R^{\text{vib}}*\left(|A_{x}{|}^{2}+|A_{y}{|}^{2}\right)\right]A_{x}\right.\right.\\ & \;+\left.\left.\left[R^{\text{rot}}*\left(4|A_{x}{|}^{2}-2|A_{y}{|}^{2}\right)\right]A_{x}\right.\right.\\ & \;+\left.\left.3\left[R^{\text{rot}}*\left(A_{x}A_{y}^{*}+A_{x}^{*}A_{y}\right)\right]A_{y}\right]\right\}. \end{array}$$ (32b)

### B. Analysis of vector Raman gain

To understand the dynamics of the vector Raman response and the underlying competition among different scattering processes, we derive and interpret the vector Raman gain, just as was done previously in the scalar case. The vector field $\vec{A}=A_{1}{\hat{e}}_{1}+A_{2}{\hat{e}}_{2}$ is assumed to be

$$A_{1}\left(z,t\right)=A_{1}^{S}\left(z,t\right)e^{i\left(\beta_{1}^{S}z-\omega_{1}^{S}t\right)}+A_{1}^{AS}\left(z,t\right)e^{i\left(\beta_{1}^{AS}z-\omega_{1}^{AS}t\right)},$$ (33a)

$$\begin{array}{ll} A_{2}\left(z,t\right) & =A_{2}^{P}\left(z,t\right)e^{i\left(\beta_{2}^{P}z-\omega_{2}^{P}t\right)}+A_{2}^{S}\left(z,t\right)e^{i\left(\beta_{2}^{S}z-\omega_{2}^{S}t\right)}\\ & \;+A_{2}^{AS}\left(z,t\right)e^{i\left(\beta_{2}^{AS}z-\omega_{2}^{AS}t\right)}, \end{array}$$ (33b)

where the pump is polarized along ${\hat{e}}_{2}$. To simplify the analysis, we consider only the most-widely used circular and linear bases. Assuming weak pump depletion, dominant terms include two factors of $A_{2}^{P}$ or combinations of its complex conjugate [$A_{2}^{P}A_{2}^{P}$, $A_{2}^{P}{\left(A_{2}^{P}\right)}^{*}$, or ${\left(A_{2}^{P}\right)}^{*}{\left(A_{2}^{P}\right)}^{*}$]. Inspection of Eq. (32) reveals that the governing equations of the Stokes/anti-Stokes waves, $A_{i}^{k}$ (*i* = 1 or 2, *k* = *S* or *AS*), contain only terms with $A_{2}^{P}A_{2}^{P}A_{1}^{*}$ and $A_{2}^{P}{\left(A_{2}^{P}\right)}^{*}A_{1}$ for the evolution of $A_{1}^{k}$, while they contain only |*A*_2_|^2^*A*_2_ for the evolution of $A_{2}^{k}$. Thus, the pump transfers energy only to Stokes and anti-Stokes waves that both have the same polarization as the pump or both have the orthogonal polarization to the pump. For example, an *x*-polarized pump does not generate or couple with *x*-polarized Stokes and *y*-polarized anti-Stokes simultaneously through SRS or FWM.

Since the evolutions of Stokes/anti-Stokes waves co-polarized with the pump have been thoroughly investigated in the scalar situation, here we examine the cross-polarized case. From Eq. (33), the coupled equation of the Stokes wave $A_{1}^{S}$, cross-polarized with the pump $A_{2}^{P}$, is

$$\begin{array}{ll} \partial_{z}A_{1}^{S}\left(z,t\right) & =i\omega^{S}\kappa^{S}\left\{\kappa_{e}^{S}\left[\kappa_{1}|A_{2}^{P}{|}^{2}A_{1}^{S}+\kappa_{2}e^{i△\beta z}{\left(A_{2}^{P}\right)}^{2}{\left(A_{1}^{AS}\right)}^{*}\right]\right.\\ & \left.\;+\left(r_{1}R_{a;1}+r_{2}R_{b;1}\right)A_{1}^{S}\right.\\ & \left.\;+r_{3}R_{b;2;P,P^{*},S}+r_{4}e^{i△\beta z}R_{b;2;P,P,AS^{*}}\right.\\ & \left.\;+\left[r_{3}\Gamma_{12}^{b\left(\Omega <0\right)}+r_{4}e^{i△\beta z}{\left(\Gamma_{12}^{b\left(\Omega >0\right)}\right)}^{*}\right]A_{2}^{P}\right\}, \end{array}$$ (34)

where again *β*_(0)_ = *β*_(1)_ = 0. The Langevin function consists of positive- and negative-frequency parts $\Gamma_{12}^{b}={\tilde{\Gamma }}_{12}^{R_{b}}/2=\Gamma_{12}^{b\left(\Omega <0\right)}e^{i\left(-△\beta^{S}z+△\omega t\right)}+\Gamma_{12}^{b\left(\Omega >0\right)}e^{i\left(-△\beta^{AS}z-△\omega t\right)}$. $R_{a;1}$ and $R_{b;2;i,j,k}$ are defined similarly to Eq. (8) with the subscripts *P* and *S*/*AS* corresponding to $A_{2}^{P}$ and $A_{1}^{S/AS}$, respectively. The pump is governed by

$$\partial_{z}A_{2}^{P}\left(z,t\right)=i\omega^{P}\kappa^{P}\left[\kappa_{e}^{P}\kappa_{3}{\left|A_{2}^{P}\right|}^{2}+\left(r_{5}R_{a;1}+r_{6}R_{b;1}\right)\right]A_{2}^{P}.$$ (35)

For a circularly polarized field, $\kappa_{1}=\frac{4}{3}$, *κ*_2_ = 0, *r*_1_ = 1, $r_{2}=\frac{1}{2}$, *r*_3_ = 1, *r*_4_ = 0, $\kappa_{3}=\frac{2}{3}$, *r*_5_ = 1, and $r_{6}=\frac{1}{2}$; for a linearly polarized field, $\kappa_{1}=\frac{2}{3}$, $\kappa_{2}=\frac{1}{3}$, *r*_1_ = 1, *r*_2_ = 0, $r_{3}=\frac{1}{2}$, $r_{4}=\frac{1}{2}$, *κ*_3_ = 1, *r*_5_ = 1, and *r*_6_ = 1. The process used to derive the scalar Raman gain yields the steady-state cross-polarized Raman gain,

$$g_{\text{ss}}^{\text{cross}-\text{circular}}=\kappa \omega^{S}|Im\left[R_{b;\text{ss}}\right]||A_{2}^{P}{|}^{2},$$ (36a)

$$\begin{array}{ll} g_{\text{ss}}^{\text{cross}-\text{linear}} & =\frac{1}{2}\kappa \omega_{R}\;Im\left[R_{b;\text{ss}}\right]|A_{2}^{P}{|}^{2}\\ & \;+\frac{1}{2}\left|\text{Re},\left[\sqrt{\left(a_{ss;+}-△\beta \right)\left(a_{ss;-}+△\beta \right)}\right]\right|, \end{array}$$ (36b)

where $R_{b;\text{ss}}$ is defined analogously to Eq. (9b) for the anisotropic Raman response function *R*_*b*_, and

$$\begin{array}{ll} a_{\text{ss};\pm } & =2\kappa \left\{\sqrt{\omega^{S}\omega^{AS}}\left(\frac{R_{b;\text{ss}}}{2}+\frac{\kappa_{e}}{3}\right){\left|A_{2}^{P}\right|}^{2}\right.\\ & \;\left.\pm \omega^{P}\left[\left(\frac{R_{b;\text{ss}}}{2}-\frac{\kappa_{e}}{3}\right){\left|A_{2}^{P}\right|}^{2}-R_{b;1}\right]\right\}. \end{array}$$ (37)

The steady-state cross-linearly polarized Raman gain asymptotically approaches 12κωSImRb;ssA2P2 as |△*β*| becomes large [Eq. (36b)], half of what can be achieved in cross-circularly polarized Raman gain [Eq. (36a)]. Unlike co-polarized situations where both isotropic and anisotropic Raman responses play an important role in the co-polarized Raman gain, only the anisotropic Raman response contributes to cross-polarized Raman gain.

**FIG. 13.**
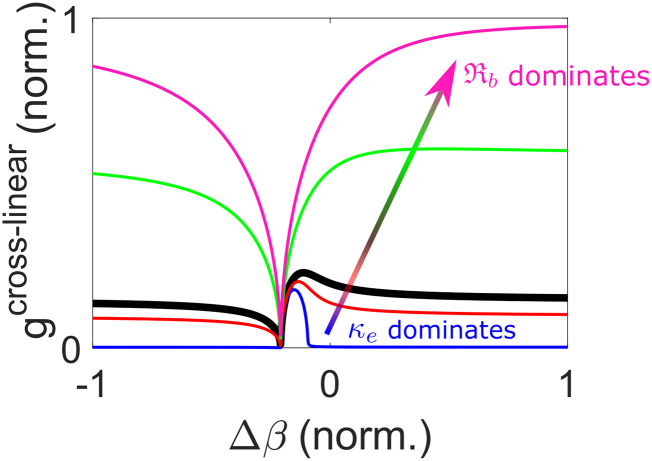
Raman gain shape *g*^cross-linear^ [Eq. (38)] vs wave-vector mismatch △*β*. The arrow indicates the trend of increasing ImRb;ss2 (blue → red → black → green → pink) with fixed $\text{Re}\left[\frac{\kappa_{e}}{3}+\frac{R_{b;\text{ss}}}{2}\right]$. The thick black line is the Raman gain with equal magnitudes of real and imaginary parts of the quantity $\left(\frac{\kappa_{e}}{3}+\frac{R_{b;\text{ss}}}{2}\right)$.

To gain insights into Eq. (36b), we assume that *ω*_*R*_ ≪ *ω*^*P*^ to obtain

$$\begin{array}{ll} g_{\text{ss}}^{\text{cross}-\text{linear}} & =\frac{1}{2}\kappa \omega_{R}\;Im\left[R_{b;\text{ss}}\right]|A_{2}^{P}{|}^{2}\\ & \;+\frac{1}{2}\left|\text{Re},\left[\sqrt{\left[4\kappa \omega^{P}\left(\frac{\kappa_{e}}{3}+\frac{R_{b;\text{ss}}}{2}\right)|A_{2}^{P}{|}^{2}-△\beta^{\prime }\right]△\beta^{\prime }}\right]\right|, \end{array}$$ (38)

where $∆\beta^{\prime }=∆\beta +2\kappa \omega^{P}\left(\frac{2\kappa_{e}}{3}{\left|A_{2}^{P}\right|}^{2}+R_{b;1}\right)$. The gain shape and its evolution with varying imaginary part of $R_{b;\text{ss}}$ are basically the same as those found in the scalar scenario (Fig. 2), but translated to negative △*β* (Fig. 13). Perfect Raman gain suppression occurs at $△\beta =-2\kappa \omega^{P}\left(\frac{2\kappa_{e}}{3}{\left|A_{2}^{P}\right|}^{2}+R_{b;1}\right)=-2\kappa \omega^{P}\left(\frac{2\kappa_{e}}{3}+\frac{R_{b}^{\text{coeff}}}{\omega_{R_{b}}}\right){\left|A_{2}^{P}\right|}^{2}$, which depends on pump wavelength and peak power.

**FIG. 14.**
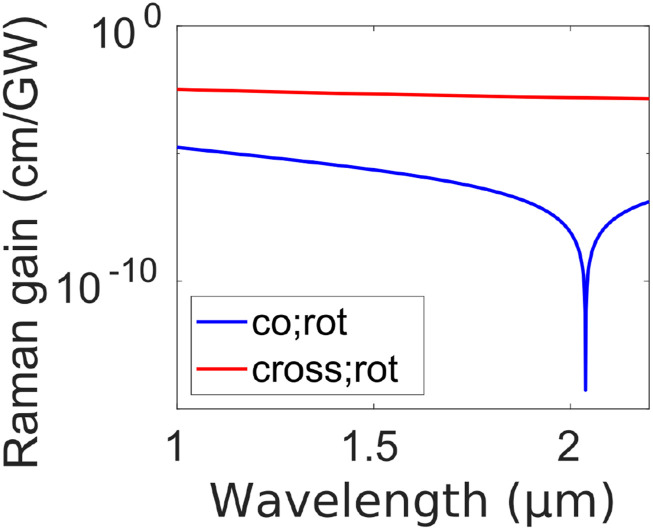
Transient S(1) rotational Raman gain of H_2_ at the pulse trailing edge for co-circularly polarized [Eq. (14)] and cross-circularly polarized [Eq. (40b)] pump and Stokes waves. A 1.5-mJ and 1-ps pulse is launched into a capillary under the same conditions as in Fig. 4. To calculate the Raman gain, the Raman-induced index change must include contributions from all Raman responses in the sum $\sum_{u}\frac{R_{u}^{\text{coeff}}}{\omega_{R_{u}}}$, which appears in $R_{1}$ and $\text{Re}\left[R_{\text{tr}}\right]$, while only one is required in $Im\left[R_{\text{tr}}\right]$ due to the narrowband feature of Raman response functions. Suppression of the co-polarized gain occurs at 2.04 *μ*m, where △*β* = 0. The ratio of the gain magnitudes is 90 at 1030 nm.

The Raman gain for the cross-circularly polarized case behaves distinctly from all other cases: it is unaffected by FWM [Eq. (36a)] because only phase-modulation terms, $O\left({\left|A_{2}^{P}\right|}^{2}A_{1}^{S}\right)$, appear in its evolution equation. In contrast to the other Raman gains, cross-circularly polarized Raman generation cannot be FWM-suppressed. This effect has been experimentally observed^117,118,121^ and modeled with a different theoretical formalism that considers only the rotational Raman response.^116,120^ In gases, wave-vector mismatch is usually small and the Raman gain is generally reduced substantially. Without a means to suppress it, the cross-circularly polarized Raman gain can be 100 times stronger than the Raman gain for other combinations of polarizations (Fig. 14).

With the Raman-gain formulas, we can calculate the ratio of Raman gains for different combinations of pump and Stokes polarizations, with negligible FWM. Although the actual Raman gain is affected by the parametric gain-suppression effect, we can gain physical intuition by knowing the approximate gain values without the suppression effect (i.e., at huge wave-vector mismatch). For co-polarized SRS, the Raman gain without FWM $g^{\left(|△\beta |\gg 1\right)}\propto |Im\left[R\right]|$ [Eqs. (10) and (14)], while for cross-polarized SRS, the ratio of Raman gains without FWM g+−(|△β|≫1):gxy(|△β|≫1)=ImRb:12ImRb [Eq. (36)]. As a result, with Eq. (32), we obtain

$$\begin{array}{ll} & g_{++}^{\left(|△\beta |\gg 1\right)}:g_{xx}^{\left(|△\beta |\gg 1\right)}:g_{+-}^{\left(|△\beta |\gg 1\right)}:g_{xy}^{\left(|△\beta |\gg 1\right)}\\ & \;=\left|\text{Im}\left[R^{\text{vib}}+R^{\text{rot}}\right]\right|:\left|\text{Im}\left[R^{\text{vib}}+4R^{\text{rot}}\right]\right|\\ & \;:6\left|\text{Im}\left[R^{\text{rot}}\right]\right|:3\left|\text{Im}\left[R^{\text{rot}}\right]\right|. \end{array}$$ (39)

The ratio of rotational Raman gains is 1:4:6:3, which is consistent with prior works.^118,119^ This consistency also supports the newly derived rotational Raman response [Eq. (31)].

For the transient regime, we can obtain the cross-polarized Raman gain by substituting $R_{b;\text{tr}}$ for $R_{b;\text{ss}}{\left|A_{2}^{P}\right|}^{2}$,

$$g_{\text{tr}}^{\text{cross}-\text{linear}}=\frac{1}{2}\kappa \omega_{R}\;Im\left[R_{b;\text{tr}}\right]+\frac{1}{2}\left|\text{Re},\left[\sqrt{\left(a_{\text{tr};+}-△\beta \right)\left(a_{\text{tr};-}+△\beta \right)}\right]\right|,$$ (40a)

$$g_{\text{tr}}^{\text{cross}-\text{circular}}=\kappa \omega^{S}\left|I,m,\left[R_{b;\text{tr}}\right]\right|,$$ (40b)

where

$$\begin{array}{ll} a_{\text{tr};\pm } & =2\kappa \left[\sqrt{\omega^{S}\omega^{AS}}\left(\frac{R_{b;\text{tr}}}{2}+\frac{\kappa_{e}}{3}{\left|A_{2}^{P}\right|}^{2}\right)\right.\\ & \left.\;\pm \omega^{P}\left(\frac{R_{b;\text{tr}}}{2}-\frac{\kappa_{e}}{3}{\left|A_{2}^{P}\right|}^{2}-R_{b;1}\right)\right]. \end{array}$$ (41)

Overall, the gain behaves similarly to the steady-state gain but with the differences pointed out in the scalar case, such as the varying gain shape throughout the pulse and the coherent properties.

**FIG. 15.**
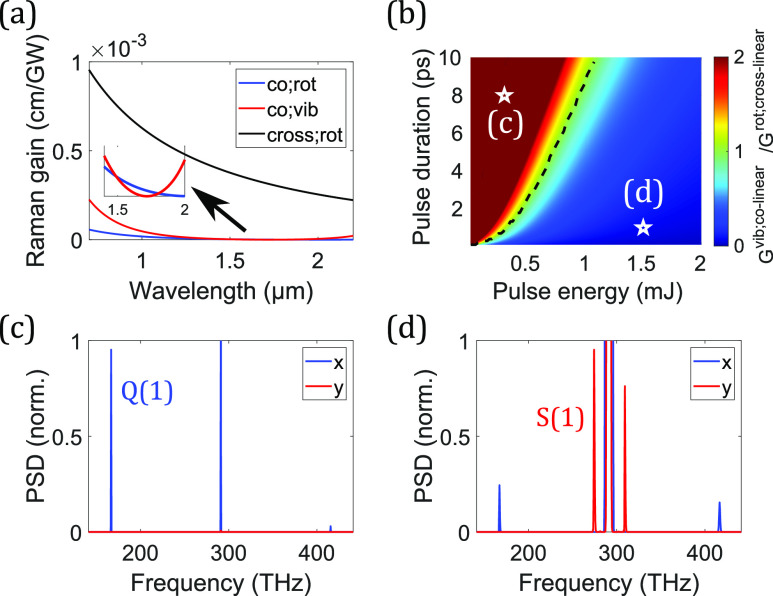
Vector aspects of Raman gain. (a) Transient Raman gain at the pulse trailing edge for S(1) rotational and Q(1) vibrational SRS co-polarized with a linearly polarized pump [Eq. (14)], as well as for S(1) rotational SRS cross-polarized with it [Eq. (40a)]. An *x*-polarized, 1.5-mJ and 1-ps pulse is launched into a capillary filled with H_2_ to a pressure of 20 bar. The inset shows the variation of co-polarized Raman gains. (b) Ratio of vibrational transient Raman gain to cross-polarized rotational transient Raman gain at 1030 nm. The vibrational and rotational gains are equal along the black dashed line. Stars indicate conditions where (c) the vibrational SRS (0.3 mJ, 8 ps) or (d) the cross-polarized rotational SRS (1.5 mJ, 1 ps) dominate. PSD: power spectral density (spectral intensity) of the pulse. The lengths of the capillary in (c) and (d) are 1 m and 10 cm, respectively, chosen to produce enough Raman generation for visualization.

Cross-polarized SRS can play an important role in experiments, with behavior that deviates from the predictions of a scalar model. As an example, for a 300-*μ*m-core capillary, filled with H_2_ to 20-bar pressure and exposed to a 1.5-mJ and 1-ps pump pulse at 1030 nm, the cross-linearly polarized rotational Raman gain is ten times stronger than the vibrational Raman gain [Fig. 15(a)]. This finding challenges the scalar prediction that vibrational SRS dominates. To further examine this phenomenon, we investigated the Raman gain for varying pulse duration and energy. Vibrational SRS exceeds the co-linearly polarized rotational SRS, as predicted by scalar calculations.^90^ However, the scalar calculation does not foresee the dominance of cross-polarized rotational SRS for wide ranges of pulse duration and energy [Fig. 15(b)]. The distinct Raman-suppression conditions for cross-linearly polarized (which depends on the pump peak power and wavelength) and co-polarized SRS (which occurs at △*β* = 0) underlie the possible control for dominant vibrational SRS [Fig. 15(c)] or cross-linearly polarized rotational SRS [Fig. 15(d)].

**FIG. 16.**
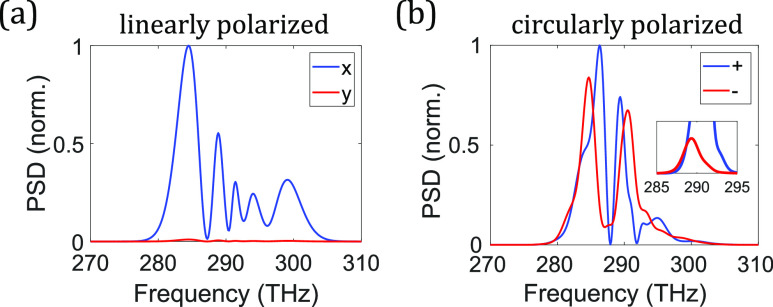
Depolarization effects in SRS. A 100-*μ*J and 300-fs pulse at 1030 nm is launched into a 2-m-long capillary, filled with N_2_ to a pressure of 3 bar. Output spectra of (a) linearly and (b) circularly polarized input pulses. PSD: power spectral density (spectral intensity) of the pulse. The inset shows the initial growth of the circularly polarized Stokes pulse in ${\hat{\varepsilon }}_{-}$ cross-polarized with the pump.

The generation of a cross-polarized Stokes or anti-Stokes wave can naturally produce depolarization of a pulse in propagation. For a linearly polarized pulse, the growth of the cross-linearly polarized Raman pulses is slow due to gain suppression. As a consequence, the linearly polarized pulse can reasonably maintain its polarization [Fig. 16(a)]. In contrast, the growth of cross-circularly polarized waves is unimpeded and is unaffected by FWM-induced gain suppression. In N_2_, with its numerous low-frequency transitions [Fig. 6(c)], SRS becomes an intrapulse effect and ultimately produces significant depolarization [Fig. 16(b)].

Vector effects are manifested not only in the Raman gain but also in nonlocal interactions mediated by phonon waves. The vector nature of the interaction offers a new degree of freedom, the polarization, for controlling Raman generation through phonon amplification or absorption processes [Eq. (20)]. Due to the different vector properties of vibrational and rotational SRS, selective excitation of one scattering process becomes possible. To investigate this effect, we assume that the second of the two pulses is weak and focus on its “linear” response to the phonon waves while ignoring its self-induced SRS. In addition, to simplify the analysis, the first pulse is polarized either in $\hat{x}$ or in ${\hat{\varepsilon }}_{+}$: ${\vec{A}}_{1}=A_{1,x}\hat{x}$ or $A_{1,+}{\hat{\varepsilon }}_{+}$. From Eq. (32), the Raman term of the UPPE for the second pulse ${\vec{A}}_{2}$ becomes

$$\begin{array}{ll} {\hat{N}}_{R}{\vec{A}}_{2} & =i\omega \kappa F\left[\left[R^{\text{vib}}*|A_{1,x}{|}^{2}\right]{\vec{A}}_{2}\right.\\ & \left.\;+\left[R^{\text{rot}}*\left(4|A_{1,x}{|}^{2}\right)\right]A_{2,x}\hat{x}\right.\left.+\left[R^{\text{rot}}*\left(-2|A_{1,x}{|}^{2}\right)\right]A_{2,y}\hat{y}\right], \end{array}$$ (42a)

$${\hat{N}}_{R}{\vec{A}}_{2}=i\omega \kappa F\left[\left[\left(R^{\text{vib}}+R^{\text{rot}}\right)*|A_{1,+}{|}^{2}\right]{\vec{A}}_{2}\right].$$ (42b)

**FIG. 17.**
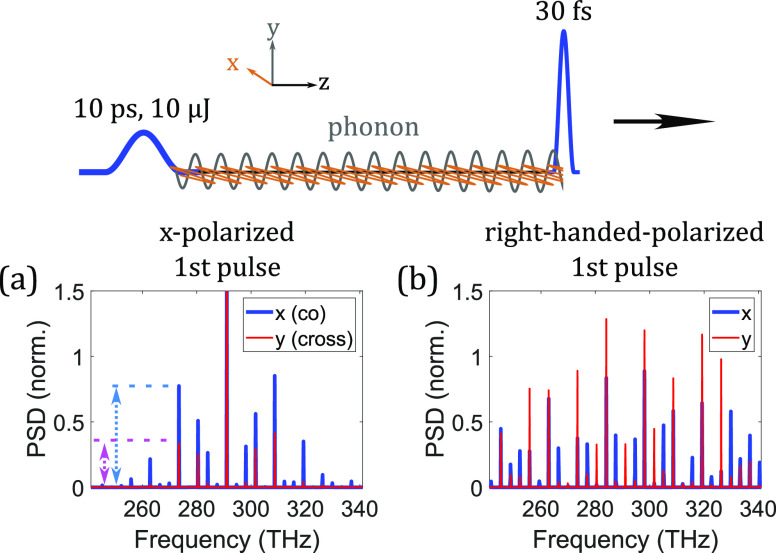
Vector effects on propagating rotational phonon waves. Two pulses are launched into a capillary filled with H_2_. The first pulse drives the S(1) rotational transition impulsively, and the second pulse scatters from the phonon waves. The energy of the first pulse is 50 *μ*J in (a) and 200 *μ*J in (b) to compensate for the four times weaker rotational SRS in circular polarization. The output spectrum of the second pulse is shown for the cases where the first pulse is (a) linearly polarized $\left(\hat{x}\right)$ or (b) circularly polarized $\left({\hat{\varepsilon }}_{+}\right)$. The second pulse is either *x*-polarized or *y*-polarized. PSD: power spectral density (spectral intensity) of the pulse. In (b), the two polarizations do not have exactly equal magnitudes because both the Stokes and anti-Stokes waves grow from noise, and the wave-vector matching is not specifically controlled [Eq. (20)].

These equations illustrate that when excited by a circularly polarized pulse, the phonon waves exhibit an isotropic behavior in relation to the second pulse [Eq. (42b)]. In contrast, if the excitation pulse is linearly polarized, the phonon waves are anisotropic for the second pulse. In this situation, the second pulse experiences a twofold increase in the strength of the rotational phonon waves, along with a *π* phase delay, when it is co-polarized as opposed to when it is cross-polarized [Eq. (42a)]. In addition, the impact of rotational phonon waves is the weakest when they are excited by a circularly polarized field. The polarization dependence that results from these effects can be exploited to drive vibrational or rotational transitions. As an explicit example, an intense ultrashort pulse was launched into a H_2_-filled capillary to impulsively excite rotational phonon waves, followed by a weak delayed pulse. The results exhibit the twofold enhancement in rotational SRS for the second pulse if the excitation pulse is linearly polarized [Fig. 17(a)], with no significant difference if it is circularly polarized [Fig. 17(b)].

## V. PERSPECTIVES

Femtosecond wavelength conversion is investigated to extend various ultrafast science applications beyond natural lasing wavelengths. As just one example, nonlinear microscopy requires high peak power at 1300 or 1700 nm for three-photon imaging.^225,226^ Raman generation in gas-filled HCF is a promising candidate for the generation of wavelengths from the ultraviolet to the mid-infrared regimes. In addition, it enables power scaling due to low nonlinearity of gases, high damage threshold, and low propagation loss.^26,227^ However, current research is mostly restricted to pulse durations from hundreds of picoseconds to a few nanoseconds, where SRS can dominate over competing nonlinearities. A relatively small number of works have targeted ultrafast (femtosecond) Raman generation.^42,48,57,68,78,86–90,128–130,228^ Detrimental competition from the Kerr nonlinearity can be avoided by appropriately stretching an ultrashort pulse in time (Fig. 4) and removing the frequency chirp from the generated Raman pulse, albeit with Raman spectral narrowing. Although femtosecond Raman generation without Raman spectral narrowing has been achieved in a two-pulse approach, it might also be possible with a simpler single-pulse approach through interference with a continuous-wave field.^53^ Additionally, Raman spectral narrowing in a single-pulse approach can be overcome by operating in the temporal regime where Raman-induced pulse compression occurs (Fig. 4). Gas-based optical parametric amplification (OPA) is another candidate for femtosecond wavelength conversion.^229,230^ Raman-enhanced SPM can potentially boost the OPA process. It also provides a new degree of freedom for controlling the process through the pulse duration, which, for example, stabilizes OPA against variations of pulse duration in a regime where increasing the duration leads to competition from the Kerr nonlinearity; the effects of lower peak power can be offset by stronger SPM enhancement.

With a deeper understanding of SRS in N_2_ and O_2_, gas-based nonlinear optics may advance toward *air photonics*, first envisioned in the terahertz regime.^231,232^ To date, most complex physical phenomena, such as phonon dynamics, have been studied in H_2_ due to its simple Raman response.^63–65,87,90,92–94,101,153,210,214,233,234^ On the other hand, investigations of N_2_ and O_2_ allow for progress toward photonics based on ambient-air-filled HCFs that can be handled the same as solid-core fibers. Prior studies in N_2_ or air include spectral broadening^75–77,127,208^ and the resulting ultrashort pulse generation,^78,79^ phonon-driven spectral control^143,200^ and transient Raman generation,^228^ soliton compression^235,236^ and self-frequency shift.^189^ Only recently has vibrational Stokes generation in N_2_ at around 1.3–1.4 *μ*m been realized in the nanosecond regime.^62^ Optimization and extension of these studies into various temporal regimes with varying pulse energies, as well as under ambient pressure, will be interesting. As an example, pulse compression in ambient-air-filled hollow-core fiber covering a broad range of pulse duration and energy can potentially offer the advantages of a waveguide (e.g., alignment-free operation and compactness) over multipass cells^237–242^ and multiplate compressors.^243–246^ Most current gas-filled pulse compressors use inert gases to avoid SRS.^239–242,247–252^ However, with specific combinations of pulse energy and duration, it will be possible to exploit SPM enhancement from SRS or to generate ultrashort spatially stabilized pulses through the formation of Raman-induced multidimensional solitary states.^79,80,82^ It will be straightforward to extend the theoretical framework of this Perspective to these other gas-based nonlinear optical platforms.

The gas pressure is a degree of freedom with HCF. This enables novel physical phenomena based on longitudinally varying pressure, which is not realizable in free-space or solid-core-fiber platforms. Self-focusing can be prevented with a negative pressure gradient, and ionization-induced defocusing can be prevented with a positive pressure gradient.^253,254^ Moreover, pressure gradients can extend the physics of tapered solid-core fiber^192,255^ to gas-filled HCF. As an example, SSFS with a positive pressure gradient exhibits several advantages over constant pressure. A flattened anomalous dispersion profile during SSFS suppresses pulse temporal broadening through the soliton area theorem *N*_*s*_ = *γ*△*t*_*p*_/|*β*_2_|, if the pulse experiences constant anomalous dispersion (|*β*_2_|) despite red-shifting, due to higher gas pressure. Increasing nonlinearity can compensate the loss of the Raman process; furthermore, it can reduce the pulse duration by maintaining *N*_*s*_ = 1. These effects combined might help push SSFS performance beyond what can be achieved with constant pressure.^68,69^ Moreover, the excitation of higher-order modes, which has been observed in recent SSFS experiments,^69^ at the fiber input can be suppressed. It is worth noting that although peak-power-induced ionization can be suppressed by gradient pressures, scaling of average power is ultimately limited by thermal effects in Raman processes,^256^ which can be resolved by reducing the repetition rate or working with pulse bursts.

Regarding technological developments, gas-filled HCF may have impact beyond research laboratories if they are pressurized and sealed, so gas-handling apparatus is not required.^257^ Such fiberized cells may enhance SRS by splicing both ends to fiber Bragg gratings to form resonant cavities.^258,259^ With continuous-wave light, 99.99% of the output light at the Stokes wavelength has been obtained,^259^ and Stokes waves at 1.7 *μ*m have been generated in H_2_^260^ or D_2_.^50^ Beyond the previous single-pass structure, an all-fiber gas Raman oscillator has been recently implemented to produce 1.8 W of continuous Stokes waves at 1693 nm through rotational SRS in H_2_.^261^ With further development of the fusion splicing technology,^262–268^ a simple and monolithic gas-based all-fiber system that supports the aforementioned SRS phenomena will be possible.

Recent years have witnessed a surge of effort in the field of quantum information science.^269–273^ Quantum frequency conversion (QFC) serves the role of connecting quantum nodes^274,275^ that operate at diverse wavelengths. It has been achieved through three-wave mixing in quadratic (*χ*^(2)^) nonlinear crystals^276–280^ and through Bragg-scattering FWM in solid-core photonic crystal fibers.^281–283^ Although solid-core fibers overcome several challenges in crystals, such as stringent phase-matching requirements and undesirable optical background, further improvements have recently been reached with a H_2_-filled hollow-core fiber.^66^ In hydrogen, long-lasting phonon waves are established through SRS, which can frequency up-convert a signal pulse with proper wave-vector matching. The theoretical framework presented above will facilitate the optimization of phonon-driven nonlocal interactions and competing Raman gains. Temporal overlap of the pump and signal pulses may be unnecessary, both frequency down- and up-conversions are possible by changing gas pressure,^284^ and controllable use of either rotational or vibrational SRS for different amounts of frequency shift is possible. To date, QFC in gases is limited to hydrogen, which offers both the huge transition frequency and simple Raman response [Fig. 6(c)];^66,284^ implementations with other gases may be desirable for various reasons. Ultimately, we foresee a promising future where ultra-tunable QFC with hollow-core fiber, filled with a wide range of gases, is achieved.

In optical quantum communication, it is crucial to create photonic quantum states with a controlled degree of entanglement and preserved coherence among the modes over long-distance transmission. Among the four degrees of freedom available for encoding a photon (polarization, two transverse spatial dimensions, and time/frequency), time-frequency encoding not only spans an unbounded high-dimensional Hilbert space but is also compatible with existing single-mode waveguide platforms. During studies of spontaneous Raman scattering, fluctuations of the generated Stokes pulse were explained as resulting from the number of excited statistically independent “coherent temporal modes,”^146,147^ or so-called “time-frequency Schmidt modes.”^285^ Recently, temporal modes have garnered increasing interest in quantum information science because they constitute an orthogonal broadband wave-packet/pulsed basis and enable the use of the time/frequency degree of freedom.^286–301^ Photon pairs are generated in various platforms, such as bulk nonlinear crystals,^288^ solid-core photonic crystal fibers,^302–306^ and tapered,^307^ birefringent,^308^ dispersion-shifted,^309–311^ or highly nonlinear^312^ fibers and waveguides.^313,314^ However, they are limited by spontaneous Raman scattering, which creates uncorrelated Raman photons.^309–311,314^ Liquid-filled^315,316^ and Xe-filled^317,318^ HCFs have been used for Raman-free photon-pair generation. As demonstrated for quantum frequency conversion by Tyumenev *et al.*,^66^ Raman interactions in gases are controllable and can exhibit (quantum-state-preserving) coherence in the transient regime. Raman interactions within gas-filled HCFs present an opportunity to avoid the imposition of adverse noisy effects on quantum communication and conversion. For example, phonon-driven processes might be considered for controllable coherent generation and conversion of temporal modes. Aforementioned ambient-air-filled or sealed pressurized HCF is a promising platform for the simplest operation. All-fiber operations based on both solid-core and hollow-core fibers might be possible with a fiberized quantum pulse gate and pulse shaper.^293,300,301^ As HCF proves its utility in achieving high-fidelity and low-latency single-photon transmission^319^ and photon-pair generation,^317,318^ the exploration of SRS in gases holds the promise of unveiling quantum applications based on temporal modes in the future.

In this Perspective, we have only considered forward SRS. However, a Stokes pulse can be generated in the opposite direction from the pump pulse. The counter-propagating Stokes pulse can not only extract energy throughout the pump pulse but also experiences strong temporal compression.^320–322^ This has been observed in several liquids,^320,321,323–325^ methane,^326–328^ and D_2_^329^ in free-space geometries. Investigations of backward SRS have also been conducted in solid-core silica fiber^330–333^ and H_2_-filled HCF.^334–338^ The transient self-similar nature of the evolution, which exhibits in forward SRS as well,^182–184^ and the corresponding periodically modulated Stokes pulse have been studied.^335,336^ Prior works have relied on a seeded process, but only recently has noise-initiated backward SRS been demonstrated to dominate the Raman process.^337^ This occurs when the pump spectral linewidth is much smaller than the Raman linewidth so that the forward and backward Raman gains are comparable. Equivalently, this corresponds to the steady-state Raman regime if the pulse is transform-limited (Fig. 1). The temporal phase profile of the backward-SRS Stokes pulse can be retained in silica fiber, as manifested by successful dechirping of the Stokes pulse from ∼23 ps to 500 fs.^333^ These observations suggest that backward SRS will have similar coherence properties as forward SRS, which motivates extension of the model presented in this Perspective to the backward situation. To date, most of the investigations of backward SRS have been confined to the steady-state Raman regime. However, the transient regime, as previously shown, can unveil diverse, unexplored, coherent physical phenomena. As demonstrated by Abdolvand *et al.* in a H_2_-filled HCF, transient amplification through backward SRS is possible.^334^ This observation raises questions about the phase relation between the pump and Stokes waves within the transient regime of backward SRS. For example, if either wave is produced by chirping a femtosecond pulse, can a femtosecond Stokes pulse be generated through backward SRS (after dechirped)? An affirmative answer could lead to more-efficient Raman generation without Raman spectral narrowing than the forward situation. Due to the dominance of forward Raman gain, it will be a challenge to demonstrate backward SRS in the transient regime, which might be overcome with gas-filled HCF.

The analysis of the vector properties of SRS in gases sheds insights on the evolution of the polarizations of the interacting fields. On the other hand, preservation of the polarization is essential to various applications, such as interferometric sensors,^339–342^ frequency metrology,^343–345^ and quantum communications.^346–350^ Hollow-core fibers with a symmetric structure can exhibit exceptional polarization purity, with up to 70-dB polarization extinction ratio,^351^ but only for linear pulse propagation. Nonlinear interactions, prevalent in ultrashort pulse propagation, can introduce significant polarization coupling through XPM, FWM, or SRS as illustrated in this Perspective. Highly birefringent hollow-core fiber preserves the polarization state by introducing a short beat length between polarization modes, effectively suppressing any polarization coupling through either linear or nonlinear interactions. Remarkably, it has achieved birefringence comparable to commercial solid-core polarization-maintaining fibers, accompanied by ultra-low loss.^352–356^ Further development of such fibers will be desirable for applications that must avoid polarization effects in Raman-active gases.

The vector physics with two polarization modes described here can be considered the initial phase of a broader exploration of multimode gas-based nonlinear optics. Multimode nonlinear effects have spurred numerous studies and potential applications. The trend began in solid-core fibers^176,357–365^ and has recently been extended to hollow-core fibers. Interested readers are referred to a recent review for multimode research.^366^ To date, multimode nonlinear effects in hollow-core fibers have been primarily studied in capillaries due to their large core size, where extreme red-shifted spectra,^78^ multidimensional solitary states,^79–82^ and few-cycle visible pulse generation^367^ have been observed. There are also recent developments in multimode hollow-core photonic crystal fibers,^355,368–373^ as well as the exploration of multimode nonlinear physics, such as circumvention of Raman gain suppression through multimode propagation.^42,103^ The unified theory covering all temporal regimes can be a starting point for understanding the potential complexities that arise from multimode interactions. As an example, we expect that the Raman-gain equations for co- and cross-polarized multimode fields will resemble those discussed here, but may exhibit unique gain-suppression relations. Intermodal SRS may play out differently in the different temporal regimes. As an example, the wave-vector-matching relations for intermodal nonlocal phonon interactions will be different from the single-mode versions. These differences may open new possibilities for controlling Raman scattering and inspire new applications.

## VI. SUMMARY

The analytic theory described in this *Perspective* relies solely on two fundamental Raman integrals [Eq. (8)]. The theory helps elucidate a wide range of Raman phenomena, including Raman gain suppression in diverse regimes, nonlinear-phase-induced Raman suppression and Raman-pulse compression, and the interplay of Raman-enhanced SPM and its transitional behavior. In addition, interactions of pulses with Raman-generated phonons in various Raman regimes can be investigated. The vector model of Raman interactions presented here facilitates the exploration of nonlinear dynamics influenced by the anisotropic nature of SRS, such as cross-polarized Raman generation, cross-polarized Raman gain, and vector nonlocal interactions. The development of the theoretical framework presented here was motivated by current directions in the interaction of ultrashort pulses with Raman-active gases, and we believe that it will be a valuable tool for future investigations in this area. We hope that this *Perspective* will be instructive for individuals who are just beginning the investigation of Raman phenomena and will also foster a deeper understanding of Raman physics in experienced researchers, all with the goal of advancing the basic science and applications of Raman scattering.

## SUPPLEMENTARY MATERIAL

See the supplementary material for supporting content.

## Data Availability

The code used in this work has been made publicly available at https://github.com/AaHaHaa/gas_UPPE. It allows for modeling not only scalar and vector but also single-mode and multimode (transverse modes + polarization modes) situations, as well as in inert (Raman-inactive) and Raman-active gases with either a constant or gradient pressure. In addition, photoionization of a single-mode scalar field is supported. Scripts to generate simulation results in this paper are also included.
